# Models of microglia depletion and replenishment elicit protective effects to alleviate vascular and neuronal damage in the diabetic murine retina

**DOI:** 10.1186/s12974-022-02659-9

**Published:** 2022-12-14

**Authors:** Kaira A. Church, Derek Rodriguez, Difernando Vanegas, Irene L. Gutierrez, Sandra M. Cardona, José L. M. Madrigal, Tejbeer Kaur, Astrid E. Cardona

**Affiliations:** 1 Department of Molecular Microbiology and Immunology, The University of Texas at San Antonio, One UTSA Circle, San Antonio, TX 78249 USA; 2 South Texas Center for Emerging Infectious Diseases, The University of Texas at San Antonio, San Antonio, TX 78249 USA; 3 Department of Pharmacology and Toxicology, Universidad Complutense de Madrid, CIBERSAM, 28040 Madrid, Spain; 4 Biomedical Sciences, School of Medicine, Creighton University, Omaha, NE 68178 USA

**Keywords:** Microglia, Depletion, Diabetic retinopathy, Repopulation, Inflammation

## Abstract

Microglia, the resident phagocytes of the retina, are believed to influence the development of retinopathy, but their exact contributions to vascular integrity and neuronal loss are unknown. Therefore, utilizing two models of microglia depletion, we aimed to deplete and repopulate microglia to clarify the contribution of microglia to neuronal loss and vascular damage in the diabetic retina in an STZ-induced model of hyperglycemia. Here, we report that 2 weeks exposure to diphtheria toxin (DTx) in diabetic CX3CR1^CreER^:R26^iDTR^ transgenic mice induced a 62% increase in Iba1^+^ microglia associated with an increase in TUJ1^+^ axonal density and prevention of NeuN^+^RBPMS^+^ neuronal loss. Conversely, diabetic PBS controls exhibited robust TUJ1^+^ axonal and NeuN^+^RBPMS^+^ neuronal loss compared to non-diabetic controls. A 2-week recovery period from DTx was associated with a 40% reduction in angiogenesis and an 85% reduction in fibrinogen deposition into the diabetic retina in comparison to diabetic PBS-treated controls. Analysis of microglia morphology and marker expression revealed that following a 2-week recovery period microglia displayed a P2RY12^+^Ly6C^–^ phenotype and high transformation index (TI) values complimented by a ramified-surveillant morphology closely resembling non-diabetic controls. In contrast, diabetic PBS-treated control mice displayed P2RY12^+^Ly6C^+^ microglia, with a 50% reduction in TI values with an amoeboid morphology. To validate these observations were due to microglia depletion, we used PLX-5622 to assess vascular and neuronal damage in the retinas of diabetic mice. Confocal microscopy revealed that PLX-5622 also induced an increase in TUJ1^+^ axonal density and prevented fibrinogen extravasation into the diabetic retina. mRNAseq gene expression analysis in retinal isolates revealed that PLX-5622-induced microglia depletion and repopulation induced a downregulation in genes associated with microglial activation and phagocytosis, *B2m*, Cx3cr1, and *Trem2*, and complement-associated synaptic pruning, *C1qa*, *C1qb*, and *C1qc*. Although the levels of microglia depletion induced with DTx in the CX3CR1^CreER^:R26^iDTR^ model and those induced with the CSF-1R antagonists are distinct, our results suggest that microglia depletion and replenishment is neuroprotective by inducing the proliferation of a homeostatic microglia pool that supports neuronal and vascular integrity.

**Supplementary Information:**

The online version contains supplementary material available at 10.1186/s12974-022-02659-9.

## Background

Diabetes mellitus, a metabolic disorder resulting in high blood glucose levels, causes systemic complications, including chronic kidney disease, fatty liver, cardiovascular disease, and vision loss due to diabetic retinopathy (DR) [1]. Although the exact mechanisms of DR pathogenesis are not fully elucidated, long-term hyperglycemia is associated with local retinal inflammation, weakened blood vessels leading to vascular damage, exudate production, hemorrhages, ischemia, and neuronal damage [2]. In addition to diet changes and insulin administration, intravitreal delivery of pharmacologic agents to target angiogenesis and surgical scarred tissue removal are common treatment modalities [2]. Unfortunately, current clinical approaches only slow vascular damage, but do not restore vision loss [2].

Maintenance and function of the blood retinal barrier (BRB) is supported by the dynamic communication between the diverse cell types comprising the retinal neurovascular unit (NVU) which include glial cells (müller glia, astrocytes, and microglia), vascular cells (endothelial cells and pericytes), and various neural cell types (amacrine cells, horizontal cells and ganglion cells). Prolonged hyperglycemia leads to BRB breakdown resulting in the three classical hallmarks of DR, (1) vascular damage, (2) neurodegeneration and (3) inflammation. As vascular damage develops in the diabetic retina, VEGF-mediated angiogenesis occurs producing poorly perfused blood vessels that escalate damage to the diabetic retina creating an ischemic environment and BRB breakdown [3, 4]. Understanding neurodegeneration in the diabetic retina may provide pathways of intervention to protect neurons as they constitute the major cell type responsible for processing and executing visual information.

Resident retinal macrophages (microglia), as phagocytes of the central nervous system (CNS), coordinate synapse development in the early postnatal retina and in adulthood support the survival and maintenance of neurons [5]. Microglia also guide blood vessel formation and maintain vascular integrity in the retina [5, 6]. In the diabetic retina, microglia rapidly respond to hyperglycemia, leukostasis and vascular leakage [7, 8]. Hyperglycemia induces vascular damage that leads to leakage of serum proteins and danger associated molecular patterns (DAMPs) into the retina, creating a loop of inflammation perpetuated by microglia proinflammatory cytokine production. However, the exact mechanisms of microglia-mediated neuroprotection or neurotoxicity in the retina remain unclear.

To clarify the contribution of retinal microglia to disease progression we sought to transiently deplete and repopulate microglia using a genetic approach, CX3CR1^CreER^:R26^iDTR^ transgenic mice, and a pharmacological approach via CSF-1R antagonist (PLX-5622) in a streptozotocin-induced diabetic model [9, 10]. Neither model resulted in complete elimination of the Iba1^+^ retinal microglia cells, but lead to significant reduction in the overall density of Iba1^+^ cells. Three-day treatment with diphtheria toxin in CX3CR1^CreER^:R26^iDTR^ mice resulted in depletion of ~ 65% of retinal microglia. Treatment with PLX-5622 for 2 weeks resulted in ablation of ~ 74% of retinal microglia, whereas 2-week treatment with diphtheria toxin (DTx) in CX3CR1^CreER^:R26^iDTR^ mice induced robust retinal EdU^+^ microglial proliferation. Detailed morphological analyses of microglia following the depletion regimens revealed morphologically ameboid cells present in the diabetic retina. However, in contrast to these phenomics, a recovery period in diabetic tissues reverted microglia back to a ramified morphology most commonly present in the non-diseased retina. We further determined that 2-week recovery period in diabetic CX3CR1^CreER^:R26^iDTR^ mice was associated with neuroprotective cues to prevent neuronal loss and decrease fibrinogen extravasation into the diabetic retina. Similar observations were noted in PLX-5622-treated CX3CR1-WT mice. Additionally, mRNAseq analysis of diabetic, PLX-5622-treated retinas revealed a retinal transcriptome with reduced expression of genes associated with DR pathogenesis, microglial activation and complement activation and synaptic pruning in microglia depleted and repopulated groups. Together this data supports the idea that microglial reprogramming can be used to enhance a homeostatic microglia cell population to regulate RGC loss and support vascular repair in the diabetic retina.

## Methods

### Mice

Due to the fact that streptozotocin does not induce consistent hyperglycemia in female mice, all experiments used male mice 6–8 weeks old. CX3CR1^CreER^ (JAX stock number: 020940; RRID:IMSR_JAX:020940), Rosa26:TdTomato, (JAX stock number: 007914; RRID:IMSR_JAX:007914), Rosa26:iDTR (JAX stock number: 007900; RRID:IMSR_JAX:007900) and CX3CR1-WT (JAX stock number: 000664; RRID:IMSR_JAX:000664) mice were purchased from The Jackson Laboratory. Rosa26:TdTomato and Rosa26:iDTR mice harbor a TdTomato (TdT) or diphtheria toxin receptor (DTR) gene, respectively, with a STOP sequence flanked by *loxP* sites. *Cre*-specific expression removes the STOP sequence allowing expression of a functional TdT or DTR gene. CX3CR1^CreER^ mice were crossed to Rosa26:TdTomato mice to generate CX3CR1^CreER^:R26^TdT^ mice. CX3CR1^CreER^:R26^iDTR^ mice were generated by crossing CX3CR1^CreER^ mice to Rosa26:iDTR mice. Mice were maintained at the Laboratory Animal Resource Center at The University of Texas at San Antonio under conventional housing conditions. All experiments were performed in accordance with National Institutes of Health guidelines and approved by UTSA-Institutional Animal Care and Use Committee.

### Two-hit model of streptozotocin-induced hyperglycemia and LPS-induced systemic inflammation

To induce hyperglycemia, mice were injected intra-peritoneally (i.p) once daily for 5 days with 60 mg/kg of streptozotocin (STZ) (Sigma Aldrich catalog number: S0130) [11, 12]. Age-matched non-diabetic controls were administered citrate buffer as a vehicle control. Blood glucose levels were measured once a week using a blood glucose monitor and animals were deemed hyperglycemic when blood glucose levels were > 250 mg/dL. To mimic systemic inflammation due to recurrent infections common in diabetic patients, all STZ-treated mice were given one daily injection of 0.08 mg/kg lipopolysaccharide (LPS) (Sigma Aldrich catalog number: L2637) for 2 days prior to euthanasia [13–16].

### Genetic microglia depletion using CX3CR1^CreER^:R26^iDTR^ mice

**Fig. 1:**
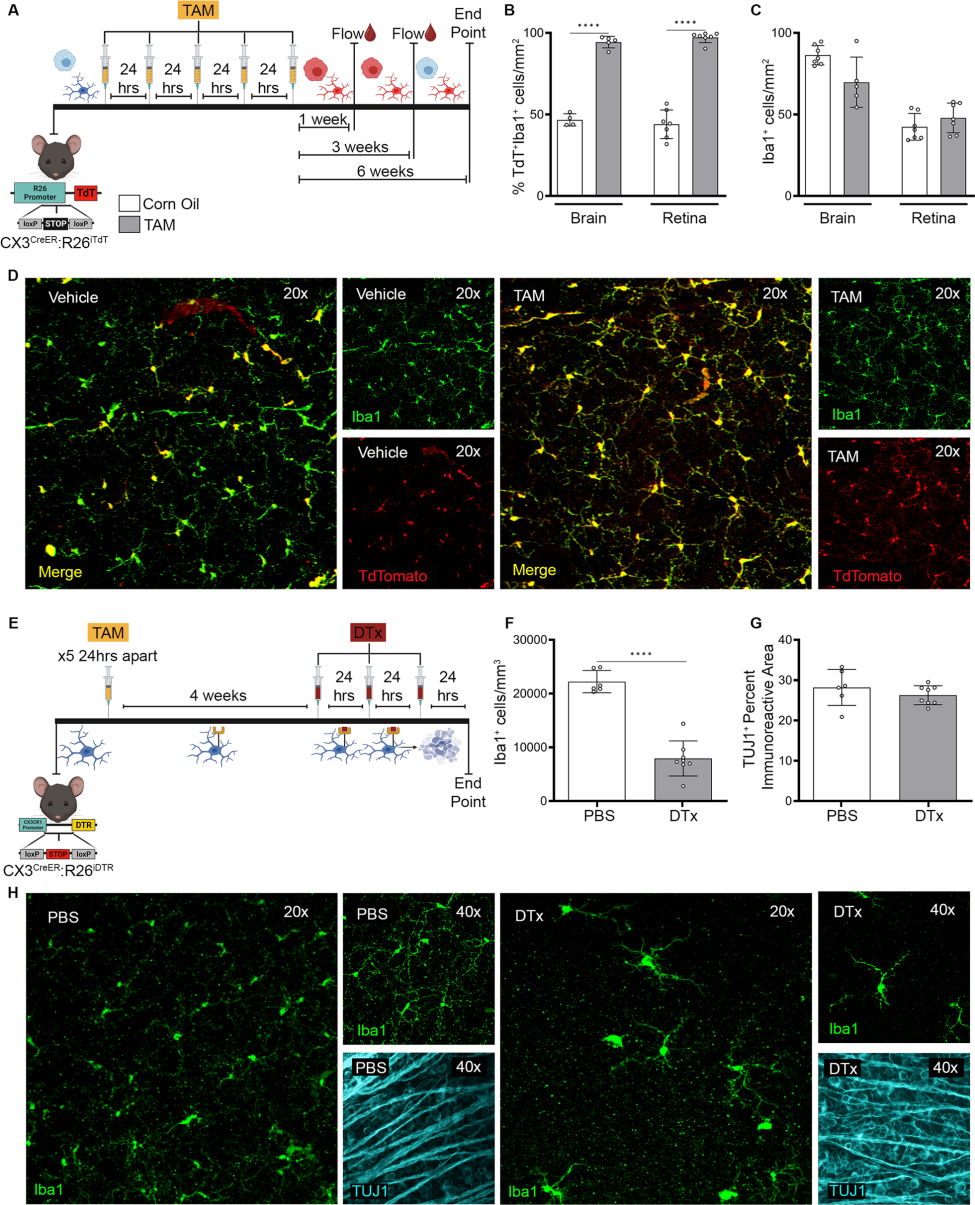
CX3CR1^CRE−ER^ expression modulated by TAM is spatially and temporally controlled in microglia. **A** Experimental design to confirm that *Cre* penetrance targets CX3CR1-expressing cells in the retina and brain without affecting peripheral *CX3CR1*-expressing immune cells. CX3^CreER^:R26^iTdT^ mice were injected once daily for 5 days with tamoxifen (TAM). One week and 3 weeks after the last TAM injection, flow cytometric analysis was performed on blood leukocytes to track the percentage of TdT^+^CD11b^+^CD45^Hi^ leukocytes. At 6 weeks post-TAM administration, tissues were collected for flow cytometric and immunohistochemical analysis. **B, C** IHC analysis of brain and retinal tissues showing the density of TdT^+^Iba1^+^ cells/mm^2^ (**B**) and Iba1^+^ cells/mm^2^ (**C**) in vehicle and TAM-treated CX3CR1^CreER^:R26^iTdT^ mice. **D** Confocal images of retinal tissues to visualize Iba1^+^ microglia (green) and TdTomato (red) in vehicle and TAM-treated CX3CR1^CreER^:R26^iTdT^ mice. **E** Experimental design to validate the feasibility of depleting CNS-resident microglia without affecting peripheral *CX3CR1*-expressing immune cells. Four weeks following *Cre* recombinase induction with TAM (after the 5^th^ TAM injection), CX3CR1^Cre−ER^:R26^iDTR^ mice were administered 25 ng/g diphtheria toxin (DTx) once daily for 3 days. Tissue collection occurred 24 h after the last DTx injection. Control mice received PBS instead of DTx. **F, G**, Quantification of Iba1^+^ cells/mm^3^ (**F**) and TUJ1^+^ percent immunoreactive area (**G**) in the retinas of PBS and DTx treated CX3CR1^CreER^:R26^iDTR^ mice. **H** Confocal images of the retina for Iba1^+^ (green), and TUJ1^+^ (white). Data represent mean ± SD, *n* = 4–7 mice per group where each dot represents an individual mouse. **P < 0.01, ****P < 0.0001 using Student’s *t*-test, with Welch’s correction

**Fig. 2:**
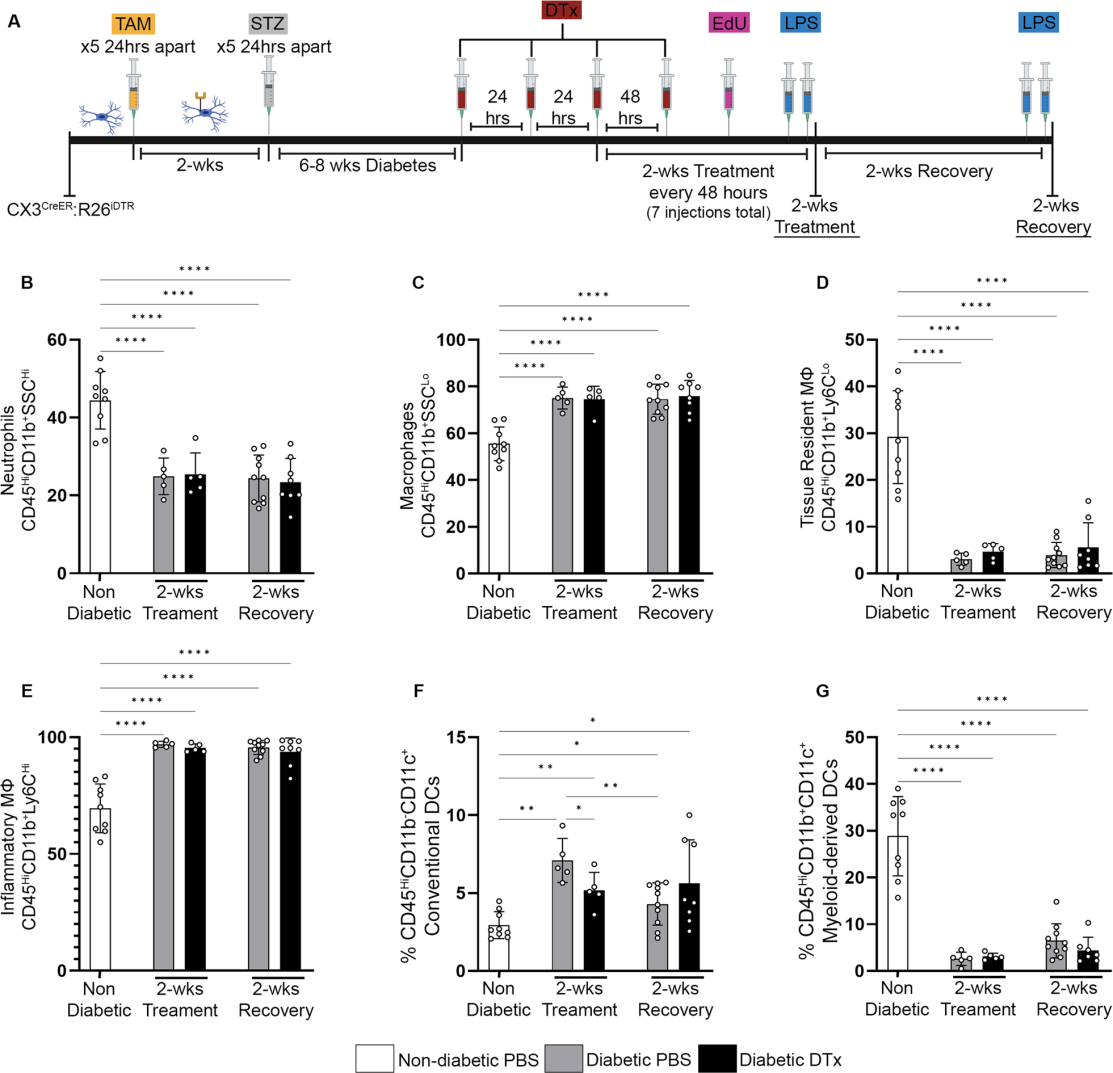
CX3CR1^CRE−ER^ expression modulated by TAM does not alter peripheral immune profile in diabetic CX3CR1^Cre−ER^:R26^iDTR^ mice. **A** Experimental design for 2-week DTx treatment in diabetic CX3CR1^Cre−ER^:R26^iDTR^ mice. Diabetes was induced via streptozotocin (STZ) in CX3CR1^CreER^:R26^iDTR^ mice 2 weeks after the last TAM injection. At 6–8 weeks of diabetes, mice received 3 daily doses of 25 ng/g DTx, followed by 1 dose of 25 ng/g DTx every 48 h for a total of 2 weeks. Tissues were collected immediately after the 2-week DTx treatment or after a 2-week recovery period. Control diabetic mice were administered PBS instead of DTx. **B–G** Flow cytometric quantification of CD45^Hi^CD11b^+^SSC^Hi^ neutrophils (**B**), CD45^Hi^CD11b^+^SSC^Lo^ macrophages (**C**), CD45^Hi^CD11b^+^Ly6C^Lo^ tissue resident macrophages (**D**), CD45^Hi^CD11b^+^Ly6C^Hi^ inflammatory macrophages (**E**), CD45^Hi^CD11b^–^CD11c^+^ conventional dendritic cells (**F**) and CD45^Hi^CD11b^+^CD11c^+^ myeloid-derived dendritic cells (**G**). Data show mean ± SD, *n* = 4–9 mice per group where each dot represents an individual mouse. *P < 0.05, **P < 0.01, ****P < 0.0001 using Student’s *t*-test, with Welch’s correction

*Cre recombinase induction*. Non-diabetic CX3CR1^CreER^:R26^TdT^ and CX3CR1^CreER^:R26^iDTR^ mice at 6 weeks of age, were given an injection of 165 mg/kg tamoxifen (TAM) (Sigma Aldrich catalog number: T5648) once a day for 5 days and age-matched controls were administered corn oil [17]. *3-day diphtheria toxin treatment* (Fig. 1E). To deplete microglia, 1 month after the last TAM injection, CX3CR1^CreER^:R26^iDTR^ mice were treated with PBS as a control or 25 ng/g diphtheria toxin (DTx) (Sigma Aldrich catalog number: D0564) once daily for 3 days. Tissues were collected 24 h after the last DTx injection. *Two-week diphtheria toxin treatment* (Fig. 2A). Two weeks following the last TAM injection, diabetes was induced in CX3CR1^CreER^:R26^iDTR^ mice and at 6 weeks of hyperglycemia, DTx was injected once daily for 3 days followed by daily injections (25 ng/g) once every 48 h for two consecutive weeks until 8 weeks of hyperglycemia [17]. In total, mice received 10 DTx injections during this 2-week treatment regimen, the first 3 injections 24 h apart, followed by 7 injections 48 h apart. PBS and DTx treated mice were pulsed with EdU at 7 weeks of diabetes, 1 week after the depletion started to determine the frequency of newly proliferating cells by administering 1 intraperitoneal injection of EdU at 50 mg/kg. Tissues were collected at 24 h after the last DTx injection at 8 weeks of diabetes. *Two-week recovery following 2-week diphtheria toxin treatment* (Fig. 2A). A separate group of mice was euthanized 2 weeks after the 2-week diphtheria toxin treatment (6-week diabetes + 2-week diphtheria toxin + 2-week recovery). Non-diabetic (ND) groups received citrate buffer instead of STZ. ND, 8-week and 10-week diabetic controls received TAM to induce the *Cre* recombinase and PBS instead of DTx.

### PLX-5622 pharmacological microglia depletion and repopulation in CX3CR1-WT mice

*Two-week PLX-5622 induced microglia depletion*. Eight weeks following STZ-induced hyperglycemia, CX3CR1-WT mice were fed 0.12% PLX-5622 chow for 2 weeks, and tissues were collected the last day of treatment when mice had progressed to 10 weeks of hyperglycemia (Fig. 5A). *Microglia depletion and repopulation for mRNA-seq analysis* (Fig. 6A). Six weeks following STZ-induced hyperglycemia, CX3CR1-WT mice were fed 0.12% PLX-5622 (MedChem Express, catalog number: HY-114153) chow (7012, Blue, TD.200435, Envigo) for 2 weeks, and tissues were collected at 8 weeks of hyperglycemia (6-week diabetes + 2-week PLX-5622 depletion). Following the 2-week PLX-5622 microglia depletion, a separate group of mice was transitioned back to normal chow and euthanized 2 weeks after microglia depletion to assess microglia repopulation (6-week diabetes + 2-week PLX-5622 depletion + 2-week repopulation). Non-diabetic (ND) groups received citrate buffer instead of STZ. ND, 6-, 8- and 10-week diabetic non-depleted controls served as normal chow controls.

### Tissue collection

Mice were transcardially perfused with cold 1 × Hank’s balanced salt solutions (HBSS) (RRID: SCR_021689). For retinal isolation, eyes were enucleated and fixed for 20 min in 4% paraformaldehyde (PFA) and post-fixed in 1% PFA for one additional hour (Sigma Aldrich catalog number: P6148). Dissected retinas were placed in cryoprotection solution (200 mL glycerol, 200 mL 0.4 M Sorenson’s buffer and 600 mL MilliQ water) overnight at 4 °C, and the following day placed in cryostorage solution (500 mL 0.2 M PO_4_, 10 g PVP-40, 300 g sucrose and 300 mL ethylene glycol) at − 20 °C. Brain tissues were dissected and fixed in 4% PFA overnight followed by cryoprotection overnight at 4 °C. Free floating brain sections (30 μm) were generated using a freezing microtome at − 20 °C and placed in cryostorage solution at − 20 °C.

### Serum collection

Blood was collected from mice via submandibular cheek bleed into tubes with K_2_EDTA (BD Microtainer catalog number: 365974). Serum tubes were centrifuged at 2200 rcf for 20 min at 4 °C. Supernatant was isolated and 1 uL of 100X proteinase inhibitor cocktail (PIC) (Roche catalog number: 04693116001) was added to serum supernatants per 100 uL of serum to bring the final PIC concentration to 1X.

### Flow cytometry

Blood was collected from mice via submandibular cheek bleed into 1.5 mL Eppendorf tubes with 30 μL of 5000 U/mL heparin (30 μL of heparin per 500 μL of blood) (Sigma Aldrich catalog number: H3393) and lysed in water for 20 s followed by the addition of 10 × HBSS and 1 × HBSS (supplemented with 10 mM HEPES). Lysed blood was centrifuged at 4 °C for 7 min at 2200 rpm. Mononuclear cells were isolated from the brains and spinal cords as previously described using Percoll gradients [18–20]. Cellular suspensions were prepared in cell staining buffer (Biolegend catalog number: 420201), blocked and stained as previously described [21]. Antibody cocktail used to label peripheral leukocytes and CNS mononuclear cells CD11b, CD45, Zombie aqua, Ly6C and CD11c. The following cell subsets were identified using the previously stated cocktail: neutrophils (CD45^Hi^CD11b^+^SSC^Hi^), tissue resident (CD45^Hi^CD11b^+^Ly6C^Lo^) and inflammatory (CD45^Hi^CD11b^+^Ly6C^Hi^) macrophages, myeloid-derived (CD45^Hi^CD11b^+^CD11c^+^) dendritic cells (DCs), conventional DCs (CD45^Hi^CD11b^–^CD11c^+^), CNS-resident microglia (CD11b^+^CD45^Lo^P2RY12^+^Ly6C^–^) and monocyte-derived microglia (CD11b^+^CD45^Lo^P2RY12^+^Ly6C^+^) [15]. Catalog numbers for the antibodies used in the antibody cocktails to stain cells are outlined in Table 1.

**Table 1:** Catalog numbers for the antibodies used in this study

Antibodies for immunohistochemical analysis			
Antibody	Company	RRID	Concentration used
Rabbit anti-ionized calcium binding adaptor molecule-1 (Iba1)	FUJIFILM-Wako	AB_839504	1:3000
Mouse anti-neuronal nuclei (NeuN)	Millipore	AB_2298772	1:4000
Guinea Pig anti RNA-binding protein with multiple splicing (RBPMS)	Millipore	AB_2687403	1:500
Mouse anti-tubulin beta 3 (TUJ1)	Biolegend	AB_10063408	1:1000
Rat anti-glial fibrillary acidic protein (GFAP)	Invitrogen	AB_2532994	1:4000
Rat anti-platelet endothelial cell adhesion molecule (PECAM-1/CD31)	BD Biosciences	AB_393571	1:500
Rabbit anti-fibrinogen	Agilent	AB_578481	1:2000
Donkey anti-rabbit 488	Jackson ImmunoResearch Laboratories, Inc	AB_2313584	1:1000
Goat anti-mouse Cy3	Jackson ImmunoResearch Laboratories, Inc	AB_2338709	1:1000
Donkey anti-guinea pig Cy3	Jackson ImmunoResearch Laboratories, Inc	AB_2340460	1:1000
Donkey anti-rat Cy5	Jackson ImmunoResearch Laboratories, Inc	AB_2340694	1:1000
Goat anti-rabbit Cy5	Jackson ImmunoResearch Laboratories, Inc	AB_2338078	1:1000
Goat anti-rat Cy3	Jackson ImmunoResearch Laboratories, Inc	AB_2338394	1:1000
Antibodies for flow cytometry			
Target antigen (Clone)	Company	RRID	Concentration used
CD11b PE-CF594 (M1/70)	BD Bioscience	AB_11154422	1:100
CD45 Pacific Blue (30-F11)	Invitrogen	AB_1518806	1:100
CD11c PE-Cy7 (N418)	Invitrogen	AB_465552	1:50
P2RY12 APC (S16007D)	Biolegend	AB_2721469	1:100
Ly6C PE (HK1.4)	Biolegend	AB_1186132	1:30

### Immunofluorescent staining

For immunohistochemical analysis, whole retinal preparations or brain tissues were blocked overnight in 10% goat or donkey serum containing 1% Triton-X 100 at 4 °C. Tissues were incubated with primary antibodies overnight at 4 °C in blocking solution (10% goat or donkey serum containing 1% Triton-X 100) followed by 5 washes each at 5 min in PBS with 0.1% Triton-X 100. Tissues were incubated in species-specific secondary antibodies to visualize proteins of interest, rabbit anti-ionized calcium binding adaptor molecule-1 (Iba1), mouse anti-neuronal nuclei (NeuN), mouse anti-β tubulin III (TUJ1), guinea pig anti-RNA-binding protein with multiple splicing (RBPMS), rat anti-glial fibrillary acidic protein (GFAP), rat anti-pecam-1 (CD31), and rabbit anti-fibrinogen (Table 1). *EdU detection*. Retinal tissues were rinsed with 1 × PBS for 5 min followed by 2 washes in 3% BSA for 5 min. Tissues were then incubated in 0.5% triton/PBS for 20 min. Next tissues were immunolabeled following manufacturer’s instructions for the In vivo EdU Click Kit 647 (Millipore Sigma, catalog number: BCK647-IV-IM-M). Following EdU immunolabeling, tissues were stained with Iba1 as outlined above.

### Confocal microscopy and image analysis

Confocal microscopy was done using a Zeiss 710 NLO confocal microscope and 3D compositions of confocal images were generated using Imaris software v7.2 (Bitplane). Six images were obtained per ¼ retinal leaflet per mouse, 2 images at the central retina nearest the optic nerve, 2 images in the middle of the leaflet and 2 images in the outer leaflet. The quantifications shown, represent the average of these 6 images spanning the 3 aforementioned regions of the retina. For Iba1^+^cells/mm^3^, NeuN^+^ cells/mm^3^, and percent immunoreactive area of CD31, fibrinogen, TUJ1 and GFAP quantifications, the following sample sizes were used: *n* = 9 non-diabetic PBS-treated, *n* = 5 8-week diabetic PBS-treated, *n* = 5 8-week diabetic DTx-treated, *n* = 10 10-week diabetic PBS-recovery and *n* = 7–8 10-week diabetic DTx-recovery mice. For Edu^+^ cells/mm^3^ quantification, the following sample sized were used: *n* = 3 non-diabetic PBS-treated, *n* = 5 8-week diabetic PBS-treated and *n* = 5 8-week diabetic DTx-treated mice. For IHC analysis in CX3CR1-WT mice (Fig. 5), the following sample sizes were used: *n* = 9 non-diabetic normal chow, *n* = 10 10-week diabetic normal chow and *n* = 10 10-week diabetic PLX-5622 chow. To quantify Iba1^+^ microglial and NeuN^+^RBPMS^+^ neuronal cell body densities, cells were manually counted in 40 × images using the counter tool in Adobe Photoshop version 21.0.3. To quantify the percent immunoreactive area of TUJ1^+^ axons, GFAP^+^ astrocytes, CD31^+^ blood vessels and fibrinogen, using ImageJ Fiji analysis software (NIH), raw confocal images were converted to 32-bit grayscale and then a global automatic threshold was applied to each image. The percent area occupied by the automatic threshold was recorded as the percent immunoreactive area. Data were normalized by volume based on X, Y and Z coordinates (i.e., 212 µm × 212 µm × Z-stack thickness) to account for changes in confocal Z-stack thickness and images size (scale settings- distance in pixels: 1024; known distance: 206.25). For microglial morphological analyses (microglia phenomics) and to determine the transformation index (TI) of microglia, individual cells were traced using ImageJ Fiji analysis software (NIH) to determine the perimeter and area of a microglia cell and calculated using the equation: perimeter^2^/4π × area^2^ [22]. The TI was determined for 5 microglia per 40 × image from each image from the central, medial and peripheral retina from 5 mice per treatment and genotype for a total of 15 microglia quantified per animal. Values are expressed as a range from 1 to 100, with a TI value closer to 1 representing a circular, amoeboid microglial cell with fewer and/or shorter cellular processes. Higher TI values represent ramified microglial cells with extensive branching and smaller cell bodies.

### RNA isolation and mRNAseq analysis

RNA was isolated from enucleated retinas by the UT Health San Antonio Biospecimen and Translational Genomics Core Laboratory using the Qiagen RNeasy Kit (Qiagen catalog number: 74104) following tissue homogenization using Zymo Research BashingBead lysis tubes (Zymo research catalog number: S6003-50). For mRNA sequencing, quality control (QC), stranded mRNAseq library prep, and sequencing were performed by UT Health San Antonio Genome Sequencing Facility. The quality of Total RNAs was assessed by Agilent Fragment Analyzer (Agilent Technologies, Santa Clara, CA), and only RNAs with RQN > 7 were used for subsequent mRNA-seq library preparation and sequencing. Approximately 500 ng of total RNA was used for stranded mRNA-seq library preparation by following the NEB Directional mRNA-seq sample preparation guide (New England Biolabs, Ipswich, MA). The first step in the workflow involved purifying the poly-A containing mRNA molecules using poly-T oligo-attached magnetic beads. Following purification, the mRNA was fragmented into small pieces using divalent cautions under elevated temperature. The cleaved RNA fragments were copied into first strand cDNA using reverse transcriptase and random primers. This was followed by second strand cDNA synthesis using DNA Polymerase I and RNase H. Strand specificity was achieved by replacing dTTP with dUTP in the Second Strand Marking Mix (SMM). These cDNA fragments then went through an end repair process, the addition of a single ‘A’ base, and then ligation of the adapters. The products were then purified and enriched with PCR to create the final RNA-seq library. Finally, RNA-seq libraries were subjected to quantification process, pooled for cBot amplification and subsequent 100 bp paired read sequencing run with Illumina NovaSeq 6000 platform. After the sequencing run, demultiplexing with Bcl2fastq2 was employed to generate the fastq file, with the average of 30 M reads per sample. Raw reads were imported into CLC Genomics Workbench v21.0.5. Initially, adaptors were trimmed and remaining reads for each sample were mapped to the annotated mouse genome (GRCm39), followed by differential gene expression (DEG) analysis of diabetic PLX-treated and diabetic PLX-recovery mice against their diabetic controls, diabetic normal chow-treated and diabetic normal chow-recovery, respectively, using the RNA-seq analysis tools within the CLC genomics software. DEG analysis was also performed on all diabetic groups, diabetic before treatment, diabetic normal chow-treated, diabetic PLX-treated, diabetic normal chow-recovery, and diabetic PLX-recovery, against non-diabetic mice. Genes were considered differentially expressed if the FDR *P*-value was < 0.05. For data mining, DEGs were defined by a cutoff of FDR *P*-value < 0.05. Principal component analysis (PCA) plots to show sample clustering by treatment type are outlined in Additional file 1: Fig. S8 and S9.

### Statistical analyses

All analyses were conducted using GraphPad Prism v9.2 and a *P* value < 0.05 was considered statistically significant. Statistical significance is denoted as *P value < 0.05, **P value < 0.01, ***P value < 0.001 and ****P value < 0.0001. Statistical tests performed included a two-tailed parametric unpaired Student's *t* test with Welch’s correction when comparing two groups. When comparing multiple groups, a two-way ANOVA with the Tukey’s post hoc test was performed, using the treatment type as the first variable and genotype as the second variable.

## Results

### Spatial and temporal regulation of CX3CR1^CRE−ER^ by tamoxifen

We used CX3CR1^CreER^:R26^TdT^ mice to confirm that *Cre* penetrance targets CX3CR1-expressing cells and to validate the feasibility of depleting resident microglia in the retina and brain without affecting peripheral *CX3CR1*-expressing immune cells (Fig. 1A). We verified *Cre* expression prevalence (TdT expression) among blood leukocytes (TdT^+^CD11b^+^CD45^Hi^) before TAM injection, and 1, 3 and 6 weeks post-TAM administration by flow cytometry (Additional file 1: Fig. S1A). We found that less than 0.02% of blood leukocytes (CD11b^+^CD45^Hi^) were TdT^+^ prior to TAM delivery in control mice (0.02 ± 0.01047) (Additional file 1: Fig. S1C). Upon TAM administration, there was a significant increase in the percentage of CD11b^+^CD45^Hi^ TdT^+^ cells at 1 week (2.083 ± 0.6445, Student’s *t* test *P* = 0.0078) and 3 weeks (2.403 ± 0.7707, Student’s *t* test *P* = 0.0007) post-TAM from 0.02% TdT^+^ cells in vehicle-treated mice (Additional file 1: Fig. S1C). However, 6 weeks after TAM administration, a time period during which bone marrow turnover allowed repopulation of blood leukocytes, there was a significant reduction in the percentage of CD11b^+^CD45^Hi^ TdT^+^ cells in TAM-treated mice (0.21 ± 0.1805) showing less than 0.4% of red fluorescent cells (Additional file 1: Fig. S1C). The data at 6 weeks post-TAM (0.21 ± 0.1805) closely resembles the levels found in corn oil-treated controls (0.02%) (Additional file 1: Fig. S1C). These results from TdT expression analysis in CD11b^+^CD45^Hi^ peripheral leukocytes confirmed that bone marrow turnover replenishes a TdT^Neg^ population in the periphery (Additional file 1: Fig. S1C). Flow cytometry on brain microglia (CD11b^+^CD45^Lo^) showed that the proportion of TdT^+^ cells in control (33.01 ± 1.841) and vehicle groups (35.97 ± 4.479) was comparable and revealed the baseline levels of leakiness of the promoter (Additional file 1: Fig. S1E). At 6 weeks post-TAM (95.77 ± 0.8770, Student’s *t* test *P* < 0.0001) administration, greater than 95% of the microglia population was CD11b^+^CD45^Lo^TdT^+^, confirming a *Cre*-specific regulation of TdT expression in CX3CR1^+^ cells (Fig. 1E). The percentage of CD11b^+^CD45^Hi^ CNS infiltrating leukocytes was comparable among control (0.7929 ± 0.5669), vehicle (0.7629 ± 0.7024) and TAM-treated (0.7486 ± 0.4678) mice (Additional file 1: Fig. S1F). These results were also supported by immunofluorescent (IF) analyses of brain and retinal tissues from corn oil and TAM-treated mice (Fig. 1B–D). In both brain and retinal tissues, TdT^+^ cells accounted for ~ 40% of the Iba1^+^ population in corn oil-treated mice, in contrast to TAM-treated mice whose Iba1^+^TdT^+^ population accounted for 100% of the microglia population (Fig. 1B, D). TAM treatment did not alter the overall Iba1^+^ microglia density in the brain and retina (Fig. 1C). These results indicate that the CX3CR1^CreER^ strain is controllable by TAM serving as a valid model to conditionally express our gene of interest, diphtheria toxin receptor (DTR), at specified time points of disease in the retina, without targeting peripheral *CX3CR1*-expressing cells.

### Three-day DTx treatment in non-diabetic CX3CR1^CreER^:R26^iDTR^ mice reveal CNS regional differences in microglia depletion

To deplete microglia, TAM-treated CX3CR1^CreER^:R26^iDTR^ mice were injected with PBS as a control or DTx (25 ng/g of body weight) once daily for 3 days and euthanized the last day of the depletion regimen (Fig. 1E). The overall population of circulating CD11b^+^CD45^Hi^ blood leukocytes remained unaltered following DTx administration (5.364 ± 1.471), closely resembling PBS (3.938 ± 1.652) treated controls (Additional file 1: Fig. S2B, C). To assess the impact of this acute depletion regimen in neuron and microglial densities, immunofluorescent analysis was used to compare retinal and brain tissues (Fig. 1F–H and Additional file 1: Fig. S2D-H). We focused on the primary visual cortex (PVC) in brain tissues because this is the region where axons synapse from the retina through the optic nerve and the dorsal lateral geniculate [23–27]. We found that acute microglia depletion resulted in a ~ 30% reduction in the overall Iba1^+^ microglia density in the PVC and did not alter the overall NeuN^+^ neuronal coverage (Additional file 1: Fig. S2D-F). However, retinal tissues were more susceptible to microglia depletion with greater than 60% of Iba1^+^ microglia cell loss after DTx administration (Fig. 1F, G). The retinas of DTx-treated (26.26 ± 2.357) mice did not display any alterations in TUJ1^+^ axonal percent immunoreactive area compared to PBS-treated (28.2 ± 4.471) controls (Fig. 1G), nor changes in astrocyte morphology or distribution (Additional file 1: Fig. S2G-H). These results reveal that this model allows successful depletion of microglia in the murine retina without depleting *CX3CR1*-expressing peripheral immune cells or eliciting acute neurotoxic effects in CNS tissues.

### CX3CR1^CRE−ER^ expression modulated by TAM does not alter peripheral immune microenvironment

Next, CX3CR1^CreER^:R26^iDTR^ mice (Fig. 2A) were analyzed after a 2-week DTx treatment and after a 2-week recovery, time points at which mice were diabetic for 8 and 10 weeks, respectively. Non-diabetic (ND), 8-week diabetic and 10-week diabetic PBS-treated groups were used as controls. We assessed peripheral immune cells and found that DTx treatment in diabetic mice did not alter the frequencies of neutrophils (CD45^Hi^CD11b^+^SSC^Hi^), tissue resident (CD45^Hi^CD11b^+^Ly6C^Lo^) and inflammatory (CD45^Hi^CD11b^+^Ly6C^Hi^) macrophages, or myeloid-derived (CD45^Hi^CD11b^+^CD11c^+^) dendritic cells (DCs) when compared to PBS-treated diabetic controls (Fig. 2B–E, G) (gating strategy Additional file 1: Fig. S3A). However, changes were detected when comparing non-diabetic and diabetic mice to include a decrease in neutrophils, tissue resident macrophages, myeloid DCs, and an increase in inflammatory macrophages in diabetic mice (Fig. 2B–E, G). A significant increase in the CD45^Hi^CD11b^–^CD11c^+^ conventional DCs population was observed when comparing ND and 8-week diabetic groups, but this population returned to baseline levels at 10 weeks of diabetes (Fig. 2F). BioPlex 23-Cytokine analysis on serum samples did not detect significant differences in the presence of cytokines associated with inflammation (IL-1a, IL-1β, IL-13, IL-17, GM-CSF, IFN- γ, MIP-1α, TNF-α, IL-6 and RANTES), proliferation (IL-3, IL-5 and IL-12p40), activation and chemotaxis (CXCL1, eotaxin and CCL-2) when comparing the DTx-recovery mice to their diabetic controls (Additional file 1: Fig. S3B-D). Differences observed in these cytokines and chemokines were due to diabetes itself as 10-week diabetic mice showed significant increases in these cytokines and chemokines when compared to ND mice (Additional file 1: Fig. S3B-D). This data suggests that under inflammatory conditions, DTx treatment does not seem to alter the peripheral immune cell profile, nor the cytokine profile environment in the periphery.

### Microglia proliferate in the retina and exhibit a ramified-like morphology following diphtheria toxin treatment in CX3CR1^CreER^:R26^iDTR^ mice

**Fig. 3:**
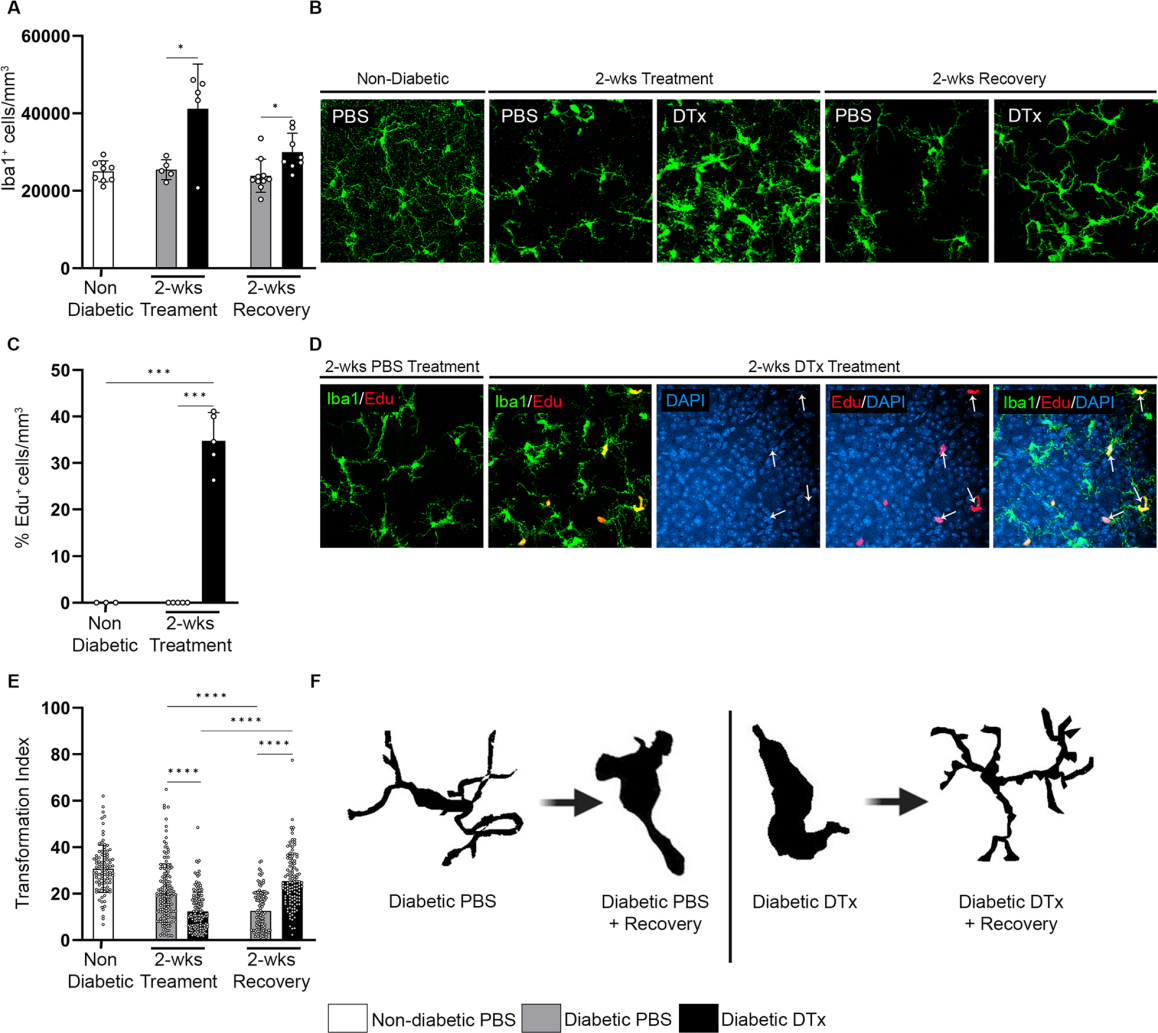
Amoeboid microglia proliferate and transition to a ramified state in the CX3CR1^Cre−ER^:R26^iDTR^ retina. **A, B** Quantification of Iba1^+^ cells/mm^3^ (**A**) and confocal images of retinas for Iba1 (green) (**B**). **C, D** Quantification of EdU^+^ cells/mm^3^ within the Iba1^+^ cell population (**C**) and confocal images of retinas for Iba1 (green), EdU (red) and DAPI (blue) in diabetic-PBS and diabetic-DTx mice (**D**). **E** Transformation index in non-diabetic, and diabetic mice PBS and DTx-treated mice, *n* = 98 to 150 microglia per group where each dot represents an individual microglia cell and bars show mean ± SD. **F** Representation of cellular tracings from transformation index quantification. Data show mean ± SD, *n* = 5–9 mice per group where each dot represents an individual mouse (**A, C**). *P < 0.05, ***P < 0.001, ****P < 0.0001 using Student’s *t*-test, with Welch’s correction

In contrast to acute DTx treatment (Fig. 1E), 2 weeks DTx treatment led to a significant increase in Iba1^+^ cells in the diabetic retina (41,157 ± 11,571) (Fig. 3A) in comparison to the PBS-treated ND (25,038 ± 2705) and diabetic controls (25,459 ± 2567, Student’s *t* test *P* = 0.0369) (Fig. 3A, B). We expected to observe a decrease in Iba1^+^ microglia cells after the 2-week DTx regimen, instead we found that ~ 35% of the retinal Iba1^+^ cells were EdU^+^ in the diabetic-DTx treated mice (34.76 ± 6.078), suggesting that long-term DTx treatment does not sustain microglia depletion (Fig. 3C, D). We next assessed microglial morphological changes by measuring their transformation index (TI) (Fig. 3E, F). In comparison to diabetic-PBS mice (20.16 ± 12.64), microglia from diabetic-DTx mice had significantly lower TIs (12.38 ± 8.34, Student’s *t*-test *P* < 0.0001) suggesting a higher activation and a phagocytic morphology. In diabetic retinas, microglia in the 2-week recovery group displayed higher TI values (25.4 ± 11.56, Student’s *t*-test *P* < 0.0001) in comparison to the corresponding diabetic-PBS control mice (12.55 ± 8.486) that displayed amoeboid microglia with low TI values (Fig. 3E, F). We also detected a significant increase in the number of Iba1^+^ cells (32,904 ± 3923, Student’s *t* test *P* = 0.0005) with significantly higher TI’s (33.78 ± 15.45, Student’s *t*-test *P* < 0.0006) in non-diabetic 2-week recovery mice in comparison to ND controls (Additional file 1: Fig. S4B, C, E, F). To validate microglial activation and to inquire about the potential origin of repopulating cells in the diabetic CNS, we did flow cytometry to distinguish CNS-resident microglia CD11b^+^CD45^Lo^P2RY12^+^Ly6C^–^ and monocyte-derived microglia CD11b^+^CD45^Lo^P2RY12^+^Ly6C^+^ (Additional file 1: Fig. S3E-H) [15]. Microglia depletion using CX3CR1^CreER^:R26^DTA^ mice demonstrated that microglial repopulation occurs from the resident pool of microglia that were resistant to depletion, and from bone marrow derived Ly6C^Hi^ monocytes that infiltrate the brain and acquire a microglia-like signature [17]. Therefore, we deemed microglia that were CD11b^+^CD45^Lo^P2RY12^+^Ly6C^+^ as monocyte-derived microglia due to their expression of Ly6C in comparison to CNS-resident microglia that do not express Ly6C. There was a significant increase in the CNS-resident CD11b^+^CD45^Lo^P2RY12^+^ microglia population in diabetic-PBS and diabetic mice after 2-week recovery (Additional file 1: Fig. S3F). There was also significant increase in monocyte-derived CD11b^+^CD45^Lo^P2RY12^+^Ly6C^+^ microglia in diabetic-PBS and diabetic-DTx mice in comparison to ND controls (Additional file 1: Fig. S3G). The frequency of monocyte-derived CD11b^+^CD45^Lo^P2RY12^+^Ly6C^+^ microglia was sustained in diabetic PBS-control mice, but a robust decrease in CD11b^+^CD45^Lo^P2RY12^+^Ly6C^+^ microglia was observed in diabetic DTx-recovery mice (Additional file 1: Fig. S3G).

### Microglia proliferation in CX3CR1^CreER^:R26^iDTR^ mice is neuroprotective and is associated with increased TUJ1^+^ axonal density

**Fig. 4:**
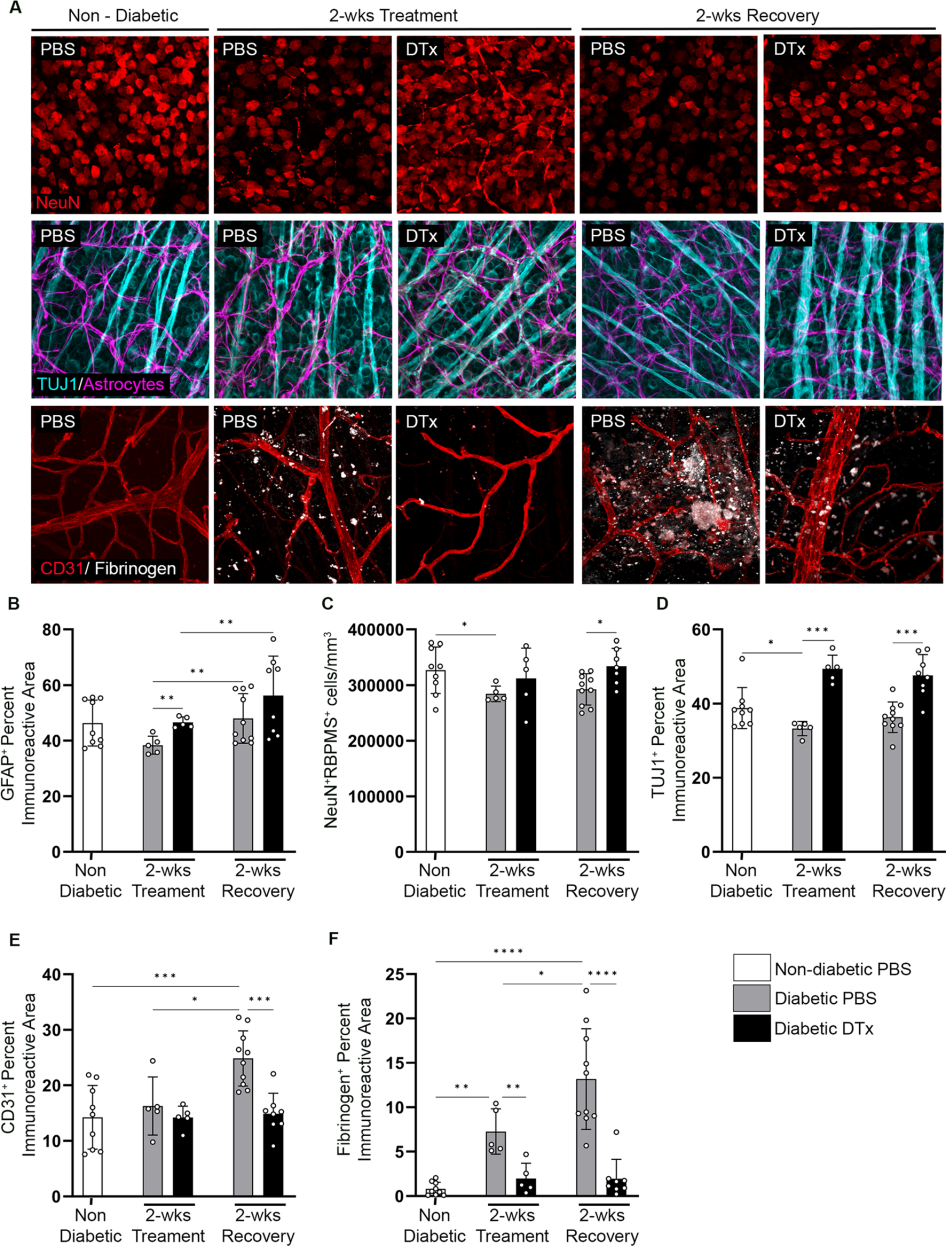
Prolonged DTx exposure and recovery prevents neurodegeneration and vascular damage in the diabetic CX3CR1^Cre−ER^:R26^iDTR^ retina. **A** Confocal images of retinal tissues stained for Iba1 (green), NeuN and RBPMS (red-top panel), TUJ1 (turquoise), GFAP (purple), CD31 (red-bottom panel) and fibrinogen (white) in non-diabetic diabetic-PBS, diabetic-DTx, diabetic PBS-recovery and diabetic DTx-recovery mice. **B–F** Quantification of retinal IHC analysis for GFAP^+^ immunoreactivity (**B**), NeuN^+^RBPMS^+^ cells/mm^3^ (**C**), and percent immunoreactive area for TUJ1 (**D**), CD31 (**E**), and fibrinogen (**F**). Data show mean ± SD, *n* = 4–9 mice per group where each dot represents an individual mouse. *P < 0.05, **P < 0.01, ***P < 0.001 ****P < 0.0001 using Student’s *t*-test, with Welch’s correction

To assess glial and neuronal responses to diphtheria toxin (same experimental design as shown in Fig. 2A), retinal tissues were stained to visualize neurons (NeuN^+^RBPMS^+^), axonal integrity (TUJ1^+^), astrocytes and Müller glia (GFAP^+^), angiogenesis (CD31^+^) and fibrinogen deposition in retinal flat mounts (Fig. 4). Diphtheria toxin treatment and recovery in non-diabetic mice did not cause changes in the number of NeuN^+^RBPMS^+^ cells (352,340 ± 28,343), TUJ1^+^ (42.29 ± 2.014) and GFAP^+^ (43.57 ± 3.759) percent immunoreactive area in comparison to ND PBS controls (Additional file 1: Fig. S4D, G-I). We observed an increase in GFAP^+^ glial cells in diabetic-DTx groups over their PBS-treated controls (Fig. 4B). However, diabetic control mice, both at 8 weeks and 10 weeks of diabetes, showed NeuN^+^RBPMS^+^ neuronal cell loss (284,504 ± 14,011 and 292,792 ± 28,127, respectively) and decreased TUJ1^+^ immunoreactivity (33.28 ± 1.928 and 36.35 ± 4.121, respectively) when compared to diabetic-DTx (NeuN^+^RBPMS^+^: 312,572 ± 54,360; TUJ1%: 49.41 ± 3.729, Student’s *t*-test *P* = 0.0001), diabetic at 2 weeks recovery (NeuN^+^RBPMS^+^: 334,078 ± 32,437, Student’s *t*-test *P* = 0.0187; TUJ1%: 47.61 ± 5.686, Student’s *t*-test *P* = 0.0005) and ND (NeuN^+^RBPMS^+^: 327,108 ± 41,863, Student’s *t* test *P* = 0.0182; TUJ1%: 38.84 ± 5.539, Student’s *t* test *P* = 0.02) mice (Fig. 4C, D). These data suggest that diphtheria toxin treatment in diabetic CX3CR1^CreER^:R26^iDTR^ mice supports a neuroprotective environment in the diabetic retina.

### Diphtheria toxin treatment in CX3CR1^CreER^:R26^iDTR^ mice correlates with decreased fibrinogen deposition in the diabetic retina

We identified vascular abnormalities by staining for CD31^+^ blood vessels and classified damaged blood vessels as those containing ruptures and fibrinogen deposits outside of the vasculature (Fig. 4A, E, F). We did not detect changes to the vasculature, nor fibrinogen deposits in the retinas of non-diabetic mice after 2-week recovery (Additional file 1: Fig. S4J-L). Ten weeks diabetic control mice displayed a significant increase in angiogenesis (24.85 ± 4.981, Student’s *t*-test *P* = 0.0006) in comparison to ND (14.27 ± 5.735) mice (Fig. 4A, E, F). Angiogenesis was not evident in 8-week diabetic-PBS (16.29 ± 5.232), diabetic-DTx (14.20 ± 2.048) or DTx-recovery (14.89 ± 3.684) mice, closely resembling ND controls (14.27 ± 5.735) (Fig. 4A, E, F). Furthermore, the vasculature in 10-week diabetic mice (PBS controls) had many vascular abnormalities with discontinuous, ruptured blood vessels, and aggregated endothelium deposits throughout the retina that colocalized with fibrinogen deposits (Fig. 4A). In addition to these vascular abnormalities, robust amounts of fibrinogen deposits were detected (13.17 ± 5.666) (Fig. 4A, F). These fibrinogen deposits in diabetic mice were initially detected in 8-week diabetic-PBS mice (7.27 ± 2.558) and only exacerbated as disease progressed to 10 weeks of diabetes (Fig. 4A, F). Conversely, diabetic DTx-treated (CD31%: 14.20 ± 2.048; fibrinogen%: 1.969 ± 1.725) and diabetic mice after 2-week recovery (CD31%: 14.89 ± 3.684; Fibrinogen%: 1.944 ± 2.198) did not show fibrinogen deposits nor vascular damage, similar to ND mice (CD31%: 14.27 ± 5.735; Fibrinogen%: 0.8082 ± 0.7421) (Fig. 4A, E, F).

### Distinct microglia depletion efficiencies and morphological changes in the CX3CR1^CreER^:R26^iDTR^ model compared to PLX-5622-treated diabetic mice

**Fig. 5:**
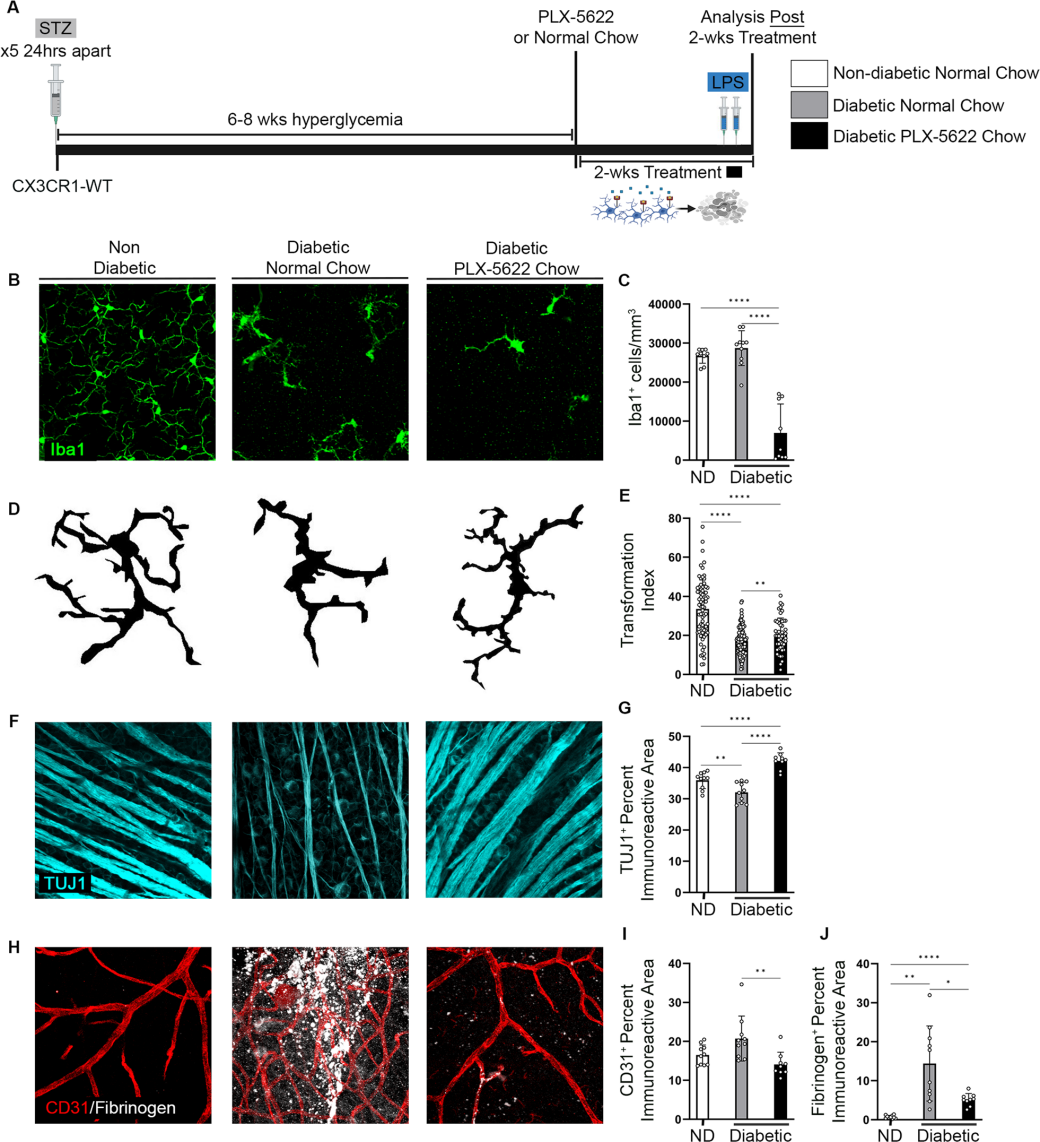
PLX-5622 treatment prevents neurodegeneration and vascular damage in the diabetic CX3CR1-WT retina. **A** Experimental design to deplete microglia in 6–8 weeks diabetic CX3CR1-WT mice with PLX-5622. Diabetes was induced via streptozotocin (STZ) in CX3CR1-WT mice and at 6–8 weeks of diabetes, mice were fed PLX-5622 chow for 2 weeks. Tissues were collected immediately following the 2-week treatment. Diabetic control mice were fed normal chow (NC). **B** Confocal images of Iba1 (green) in retinas of non-diabetic, diabetic-normal chow and diabetic PLX-5622 chow mice. **C** Quantification of Iba1^+^ cells/mm^3^. **D, E**, Representation of Iba1^+^ cellular tracings (**D**) from transformation index quantification (**E**). **F** Confocal images of TUJ1 (turquoise) in retinas of non-diabetic, diabetic-normal chow and diabetic PLX-5622 treated mice. **G** Quantification of TUJ1^+^ percent immunoreactive area. **H–J** Confocal images of retinas stained for CD31 (red) and fibrinogen (white) (**H**) and image quantification of percent immunoreactive area for CD31 (**I**) and fibrinogen (**J**). Data show mean ± SD, *n* = 8–10 mice per group where each dot represents an individual mouse (**C, G, I, J**). Data are mean ± SD, *n* = 52–150 microglia per group where each dot represents an individual microglia cell from *n* = 4–10 mice (**E**). *P < 0.05, **P < 0.01, ***P < 0.001 ****P < 0.0001 using Student’s *t*-test, with Welch’s correction

We compared the degree of microglia depletion in diabetic CX3CR1^CreER^:R26^iDTR^ mice DTx treated for 2 weeks and in diabetic CX3CR1-WT mice, PLX-5622 treated for 2 weeks (Additional file 1: Fig. S6A). Flow cytometric analysis of brain and spinal cord tissues revealed no difference in the percentage of live CD11b^+^CD45^Lo^Zombie^–^ microglia in DTx-treated CX3CR1^CreER^:R26^iDTR^ mice (94.22 ± 1.469, Student’s *t* test *P* = 0.0006) in comparison to their PBS-treated diabetic CX3CR1^CreER^:R26^iDTR^ controls (95.12 ± 0.9706) (Additional file 1: Fig. S6B). We observed a significant reduction in the percentage of live CD11b^+^CD45^Lo^Zombie^–^ microglia in PLX-5622-treated CX3CR1-WT mice (30.06 ± 4.95, Student’s *t* test *P* < 0.0001) in comparison to their diabetic, normal chow CX3CR1-WT controls (99.56 ± 0.1776) (Additional file 1: Fig. S6B). In retinal tissues, the data showed a significant increase in Iba1^+^ cells in the DTx-treated CX3CR1^CreER^:R26^iDTR^ mice (41,157 ± 11,571, Student’s *t* test *P* = 0.0345) compared to their diabetic CX3CR1^CreER^:R26^iDTR^ PBS controls (25,459 ± 2567). In PLX-5622 treated CX3CR1-WT mice (6994 ± 7422, Student’s *t* test *P* < 0.0001), we detected a robust reduction in Iba1^+^ cells in the retina of compared to their diabetic CX3CR1-WT normal chow controls (28,721 ± 4462) (Additional file 1: Fig. S6C). These results highlight the differences in the kinetics of microglia depletion with DTx and PLX-5622. Following depletion, diabetic DTx-treated CX3CR1^CreER^:R26^iDTR^ mice had a reduction in their TI (12.38 ± 8.34, Student’s *t* test *P* < 0.0001) compared to their diabetic, CX3CR1^CreER^:R26^iDTR^ PBS controls (30.73 ± 10.16), indicative of increased activation in DTx-treated mice (Additional file 1: Fig. S6D). In contrast, PLX-5622-treated diabetic CX3CR1-WT mice exhibited a ~ 20% increase in their TI (20.49 ± 8.502, Student’s *t* test *P* < 0.0001) compared to their diabetic, normal chow CX3CR1-WT controls (17.03 ± 6.729) shifting microglia to a more branched and ramified morphology (Fig. 5D, E and Additional file 1: Fig. S6D).

### Robust microglia depletion with PLX-5622 promotes an increase in TUJ1^+^ axonal density and prevents fibrinogen deposition in the diabetic CX3CR1-WT retina

Diabetes induced significant TUJ1^+^ axonal loss in diabetic mice (32.08 ± 3.225, Student’s *t* test *P* = 0.0095) in comparison to ND mice (35.95 ± 2.703) (Fig. 5F, G). PLX-5622 treatment correlated with an increase in TUJ1^+^ axonal density in diabetic mice (42.15 ± 2.59, Student’s *t* test *P* < 0.0001) in comparison to diabetic control mice (Fig. 5F, G). Consistent with previous results (Fig. 4E, F), diabetes induced angiogenesis, ruptured blood vessels and fibrinogen deposition in diabetic mice (CD31: 20.73; fibrinogen: 14.42, Student’s *t* test *P* = 0.0015) in comparison to ND mice (CD31: 16.51 ± 2.565; fibrinogen: 0.6982 ± 0.4107) (Fig. 5H–J). This pathology was ameliorated in diabetic PLX-5622-treated retinas which displayed intact vasculature with little fibrinogen extravasation into the diabetic retina (CD31: 14.1 ± 3.115, Student’s *t* test *P* = 0.007); fibrinogen: 5.141 ± 1.563, Student’s *t* test *P* = 0.0139) (Fig. 5H–J).

### Homeostatic microglia gene expression profile is associated with pharmacological microglia depletion and repopulation in diabetic CX3CR1-WT mice

**Fig. 6:**
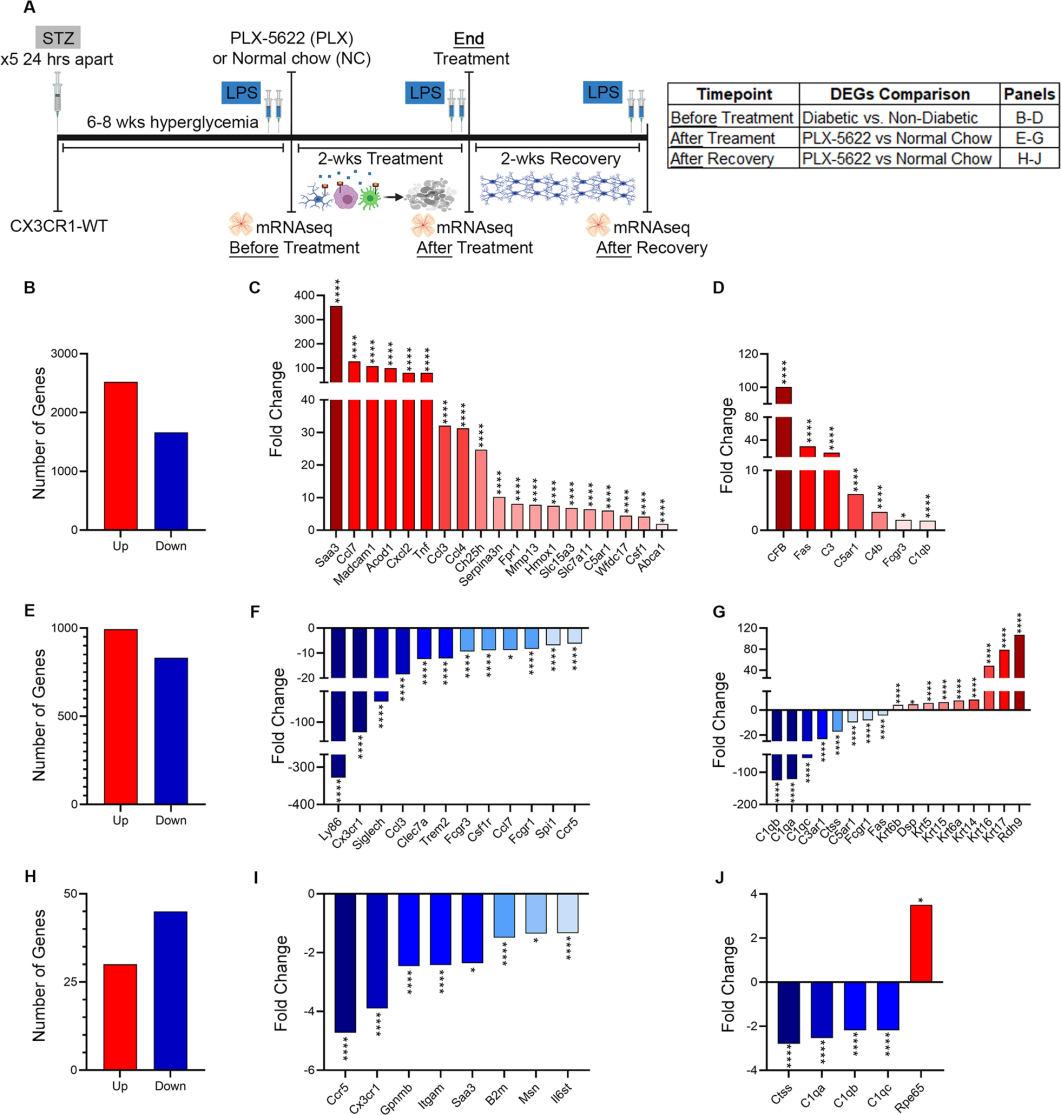
Microglia depletion and repopulation resets the diabetic retinal transcriptome to closely resemble non-diabetic controls. **A** Experimental design to pharmacologically deplete and repopulate microglia using PLX-5622 for retinal mRNAseq analysis. Six weeks following STZ-induced diabetes, CX3CR1-WT mice were fed PLX-5622 for 2 weeks, followed by a 2-week recovery period. Diabetic control mice were fed normal chow. **B–D**, Differentially expressed genes (DEGs) analysis in diabetic mice before the treatment versus non-diabetic mice for total number of DEGs (**B**), analysis of DEGs associated with DR pathogenesis and microglial activation (**C**), and complement-mediated synaptic pruning and intermediate filament organization, visual cycle and wellness (**D**). **E–G** DEGs analysis in diabetic mice after PLX-5622 treatment versus diabetic normal chow mice for total number of DEGs (**E**), analysis of DEGs associated with DR pathogenesis and microglial activation (**F**), and complement-mediated synaptic pruning and intermediate filament organization, visual cycle and wellness (**G**). **H–J** DEGs analysis in diabetic mice after PLX-5622 recovery versus diabetic normal chow mice for total number of DEGs (**H**), analysis of DEGs associated with DR pathogenesis and microglial activation (**I**), and complement-mediated synaptic pruning and intermediate filament organization, visual cycle and wellness (**J**)

We next performed mRNAseq on whole retinas from non-diabetic and diabetic mice with or without microglia depletion to probe general transcriptional changes that would give rise to the neuro- and vasculo-protective effects of transient microglia depletion and repopulation in the diabetic retina (Fig. 6A). Microglia were pharmacologically depleted in 6-week diabetic CX3CR1-WT mice using PLX-5622 and analyzed at three different timepoints: (1) at 6 weeks of diabetes (6D) to establish the retinal transcriptome prior to microglia depletion; (2) after 2 weeks microglia depletion (PLX-treatment), and (3) after 2 weeks of microglia repopulation (PLX-recovery) (Fig. 6A). At each timepoint, ND, diabetic normal chow (NC) treated, and NC recovery were included (Fig. 6A). Microglial activation and expression of disease-associated genes are highly correlated to retinal inflammation and degeneration [8]. We identified 2520 differentially expressed genes (DEGs) significantly upregulated and 1,664 DEGs significantly downregulated in diabetic mice before treatment relative to non-diabetic mice (Fig. 6B). Analysis of diabetic retinas before treatment revealed a significant increase in DEGs associated with DR pathogenesis and microglial activation (*Saa3*, *Ccl7*, *Madcam1*, *Tnf*, *Ccl3*, *Acod1*, *Cxcl2*, *Ccl4*, *Ch25h*, *Serpina3n*, *Fpr1*, *Mmp13*, *Hmox1*, *Slc15a3*, *Slc7a11*, *Wfdc17*, *Csf1* and *Abca1*), and complement activation (*CFB*, *Fas*, *C3*, *C5ar1*, *C4b*, *Fcgr3*, *C1qb*) revealing the baseline levels of inflammation prior to depletion (Fig. 6C, D). We identified 994 DEGs significantly upregulated and 831 DEGs significantly downregulated in PLX-treated relative to NC mice and 30 significantly upregulated DEGs and 45 significantly downregulated DEGs in PLX-recovery mice relative to NC-recovery mice (Fig. 6E, H). Genes associated with DR pathogenesis and microglial activation and phagocytosis (*Ccl3*, *Ccl7*, *Ccr5*, *Ly86*, *Cx3cr1*, *Siglech*, *Clec7a*, *Fcgr3*, *Csf1r*, *Fcrg1, Itgam, B2m, Msn, Il6st*) were significantly downregulated in diabetic PLX-treated and PLX-recovery mice when compared to diabetic NC mice, with the greatest fold changes seen in PLX-treated mice (Fig. 6F, I). Diabetic PLX-treated and PLX-recovery mice displayed a significant downregulation of complement-associated genes to include *C3ar1*, *Ctss*, *C5ar1*, *C1qa*, *C1qb* and *C1qc* (Fig. 6G, J).To corroborate our findings that microglia replenishment is neuroprotective in the diabetic retina, we assessed genes associated with intermediate filament organization (*Krt17*, *Krt16*, *Krt14*, *Krt15*, *Krt6a*, *Krt6b*, *Krt5* and *Dsp*), visual cycle wellness (*Rpe65* and *Rdh9*) and found that these genes were significantly upregulated in diabetic PLX-treated mice compared to diabetic NC controls (Fig. 6G, J). We also identified *GFAP*, *Claudin-1*, *Claudin-4* and *F11r* transcripts significantly upregulated in diabetic PLX-treated and PLX-recovery mice indicative of strengthening of the glial limitans (Additional file 1: Fig. S7B-E), and vascular protection (*Adamts13* and *Csf3*) (Additional file 1: Fig. S7F-G).

## Discussion

To clarify the role of microglia in DR initiation and progression, we utilized two models to deplete microglia, a spatially and temporally controlled conditional model using CX3CR1^CreER^:R26^iDTR^ mice, and a pharmacological model, PLX-5622, in *CX3CR1*-WT mice [9, 10]. Analysis of brain, spinal cord and retinal CX3CR1^CreER^:R26^iDTR^ tissues revealed CNS regional differences in the extent of microglial depletion, with greater susceptibility in the retina following acute DTx treatment. Prolonged DTx treatment induced significant Iba1^+^Edu^+^ retinal microglia proliferation, whereas PLX-5622 microglial depletion yielded robust and proportional microglial depletion in brain and retinal tissues. Among both models, we found that DTx treatment in CX3CR1^CreER^:R26^iDTR^ mice and PLX-5622 treatment in CX3CR1-WT mice correlated with reduced pathogenic angiogenesis, vascular damage, and neuronal cell loss in diabetic retinas. Furthermore, a 2-week recovery after DTx treatment in CX3CR1^CreER^:R26^iDTR^ mice, maintained these neuroprotective and vasculo-protective effects. mRNAseq analysis of PLX-5622 transient microglia depletion and repopulation illuminated potential regulatory pathways related to neuronal and vascular damage and inflammation.

Herein this study, we compared CX3CR1^CreER^:R26^iDTR^ brain and retinal tissues for the degree of microglia depletion following acute DTx treatment to address the possibility that microglia depletion can have various effects depending on the CNS compartment. In comparison to the brain, we found that the retina was more susceptible to acute microglia depletion, with greater than 60% microglia depletion in DTx-treated mice (Fig. 1E–H). We also report that PLX-5622 yields a robust amount of microglia depletion, in the brain by flow cytometric analysis (Additional file 1: Fig. S6B), and the retina by IHC analysis (Fig. 5B, C). In contrast, in the DTR model, DTx treatment did not induce any alterations in the percentage of CD11b^+^CD45^Lo^Zombie^–^ microglia in the brain and spinal cord detected by flow cytometry (Additional file 1: Fig. S6B). The striking difference in the degree of depletion could be due to the pharmacokinetics and half-life of DTx and PLX-5622, and the frequency with which these drugs are delivered. Additionally, following the 2-week DTx treatment, we observed a robust increase in Iba1^+^ cells in the diabetic CX3CR1^CreER^:R26^iDTR^ retina (Fig. 3A, B). Previous studies have shown that following depletion, microglia repopulate from a resident pool of depletion-resident microglia and from peripheral infiltrating macrophages that can acquire a microglia-like signature [17]. Flow cytometric analysis of brain and spinal cord revealed a significant increase in the percentage of Ly6C^+^P2RY12^+^ mononuclear cells in diabetic mice, with no differences in PBS versus DTx treated mice (Additional file 1: Fig. S3G). However, following a 2-week recovery, DTx-treated mice had a significant reduction in the percentage of Ly6C^+^P2RY12^+^ mononuclear cells in the brain and spinal cord (Additional file 1: Fig. S3G) and in the number of Iba1^+^ cells in the retina (Fig. 3A, B). Furthermore, analysis of DTx treatment in the non-diabetic CX3CR1^CreER^:R26^iDTR^ retina revealed a significant ~ 31% increase in Iba1^+^ cells in the non-diabetic DTx-recovery retina, and a significant ~ 89% increase (Student’s *t* test, *P* = 0.0084) in Ly6C^+^P2RY12^+^ mononuclear cells in brain and spinal cord by flow cytometry, suggesting that this increase in Iba1^+^ cells in the retina could potentially be from infiltrating monocyte-derived macrophages (Additional file 1: Figs. S4 and S5). Moreover, since acute DTx treatment induced microglial depletion and prolonged DTx exposure induced microglial proliferation, these findings raise the question if continued DTx exposure increases proliferation rates in microglia. Additional studies assessing various timepoints following DTx could provide further insights into microglia depletion and proliferation following DTx treatment.

In experimental models of DR and human diabetes, neurodegeneration has been reported to be an early indicator of DR pathogenesis as evident by a robust increase in TUNEL-positive neurons and thinning of the retinal nerve fiber layer (RNFL) [28–30]. Neurodegeneration in DR has been shown to be complement-mediated and complement-based therapies are a strong candidate for treatment of retinal degenerative diseases [31]. In models of age-related macular degeneration (AMD), C3-expressing microglia and complement deposited proteins colocalized to lesions of photo-oxidative damage perpetrating neurodegeneration and intravitreal injection of C3 small interfering RNA (siRNA) prevented neurodegeneration [32]. A downregulation in complement-associated gene clusters (*C3ar1*, *C1qb*, *C1qa, C1qc*) in diabetic PLX-5622-treated mice relates to the observations of less NeuN^+^RBPMS^+^ neuronal cell loss in CX3CR1^CreER^:R26^iDTR^ mice (Figs. 4 and 6). In addition to preserved NeuN^+^RBPMS^+^ neurons, both models, DTx treatment in CX3CR1^CreER^:R26^iDTR^ mice and PLX-5622 treatment in CX3CR1-WT, were associated with an increase in TUJ1^+^ axonal density in the diabetic retina (Figs. 4 and 5). The keratin class of intermediate filaments are the major source of neurofibrils that give axons their fundamental structure therefore we focused on keratin-associated gene clusters since [33]. PLX-5622 treatment induced an upregulation of keratin-associated gene clusters (*Krt17*, *Krt16*, *Krt14*, *Krt15*, *Krt6a*, *Krt5* and *Dsp*) (Fig. 6) substantiating our findings of increased TUJ1^+^ axonal density (Figs. 4 and 5). Although in a model of optic nerve crush, microglia depletion did not alter retinal ganglion cell degeneration and regeneration, data presented here supports other studies highlighting the neuroprotective effects of microglia depletion in retinopathy models, including autoimmune uveitis and excitotoxicity-induced neuronal cell death in which microglia depletion alleviated clinical symptoms and the degree of neuronal cell loss [34–36].

Under inflammatory conditions, reactive astrocyte production of metalloproteases and upregulation of tight junction (TJ) proteins and junctional adhesion molecules (JAMs) strengthens the glial limitans of the blood brain barrier to restrict leukocyte extravasation and adhesion to inflamed venules that exacerbate CNS lesion formation [37–39]. Additionally, astrocyte production of neurotrophic factors also aids in vascular protection and promotes neuroprotection [40–42]. Together the upregulation *GFAP*, *Claudin-1*, *Claudin-4*, *F11r*, *Adamts13* and *Csf3* (Additional file 1: Fig. S7) supports the amelioration of vascular damage and fibrinogen deposition visualized by IHC analysis of CD31^+^ blood vessels and fibrinogen (Figs. 4 and 5) in both models of microglia depletion used. IHC analysis of GFAP^+^ glial responses from astrocytes and Müller glia revealed significant increase in the GFAP^+^ percent immunoreactive area in DTx-treated mice (Fig. 4A, B). Further studies characterizing the responses from astrocytes and Müller glia could provide further insight on the effects of microglia depletion to these glial cells that are important in maintaining retinal integrity.

Uncontrolled hyperglycemia diminishes BRB integrity causing neuronal cell apoptosis and vascular damage leading to the activation of the resident professional phagocytes, microglia. Many reports have shown that diabetes results in proinflammatory, phagocytic microglial activation, characterized by an ameboid shape with truncated, retracted cellular processes and microglia-derived IL-1β and NOS2 production [12, 43, 44]. Intriguingly, DTx treatment resulted in ramified microglia with long cellular processes in diabetic mice, closely resembling non-diabetic controls (Fig. 3E, F). Additionally, diabetic PLX-5622-treated mice displayed a 20% increase in microglial TI, with increased cellular branching, in contrast to ameboid-shaped microglia in diabetic controls (Fig. 5). Consistent with these results, transcriptomic analysis (Fig. 6F, I) revealed a reduction in microglial activation associated gene clusters (*Ly86*, *Cx3cr1*, *Siglech*, *Clec7a*, *Trem2*, *Fcgr3*, *Csf1r*, *Fcrg1*) indicative that these changes in microglia morphology correlate with transcriptional reprograming. Additionally, it was shown that fibrinogen depletion using the defibrinogenating agent Ancrod, ameliorated reactive microgliosis and release of proinflammatory cytokines in the diabetic retina [45]. Complementary to these findings, depleting microglia reduced fibrinogen deposition in the diabetic retina (Figs. 4 and 5). Although our data suggest that these depletion regimens reprogram microglia to retain a homeostatic response to the diabetic environment, the length of time it takes for these repopulating microglia to obtain a proinflammatory profile is worth exploration to characterize the duration of these protective effects.

Due to lack of glucose uptake, metabolic dysregulation occurs in peripheral cells leading to the production of DAMPs and proinflammatory serum proteins [46]. This chronic, low-grade inflammation present in the periphery contributes to the retinal manifestations of disease and contributes to the loop of inflammation present in the retina. In the CD11b-HSVTK and clodronate liposome delivery models of microglia depletion, BBB/BRB damage facilitates peripheral immune cells infiltration to the CNS [47, 48]. Additionally, the use of CSF-1R inhibitors does not only target microglia, but has also been shown to deplete CCR2^+^ monocyte progenitors, bone marrow derived macrophages, hematopoietic progenitor and stem cells in the bone marrow, spleen and blood compartments [49]. Utilizing the CX3CR1^CreER^:R26^iDTR^ model of microglia depletion, we were able to successfully deplete microglia without affecting peripheral immune cell proportions in the blood (Fig. 2 and Additional file 1: Fig. S3) nor did this model of depletion alter peripheral cytokine responses (Additional file 1: Fig. S3).

Overall, this study highlights differences in the degree of microglia depletion between two commonly used models, defines depletion differences among CNS compartments and also provides a list of potential candidate gene targets to mediate microglial inflammation. This study shows that microglia depletion and replenishment ameliorates microglia-associated retinal inflammation and hallmarks of DR pathogenesis to include neuronal cell loss, pathogenic angiogenesis, vascular damage and inflammation. Microglia reprogramming was validated by morphological and transcriptomic analysis and these results support the idea that transient microglia depletion and repopulation triggers neuro- and vasculo-protective effects in the diabetic retina.

## Supplementary Information

**Additional file 1: Fig S1.** Characterizing CX3CR1^CRE−ER^ expression modulated by TAM in CX3^CreER^:R26^iTdT^ mice. (A) Experimental design to confirm that *Cre* penetrance targets CX3CR1-expressing cells in the retina and brain without affecting peripheral *CX3CR1*-expressing immune cells. CX3^CreER^:R26^iTdT^ mice were injected once daily for 5 days with tamoxifen (TAM). One week and 3-weeks after the last TAM injection, flow cytometric analysis was performed on blood leukocytes to track the percentage of TdT^+^CD11b^+^CD45^Hi^ leukocytes. At six-weeks post TAM administration, tissues were collected for flow cytometric and immunohistochemical analysis. (B) Gating strategy to identify TdT^+^CD11b^+^CD45^Hi^ blood leukocytes. (C) Graphical representation of flow cytometric quantification of TdT^+^CD11b^+^CD45^Hi^ blood leukocytes. (D) Gating strategy to identify TdT^+^CD11b^+^CD45^Lo^ microglia in brain and spinal cord tissues. E–F, Graphical representation of flow cytometric quantification of TdT^+^CD11b^+^CD45^Lo^ microglia (E) and TdT^+^CD11b^+^CD45^Hi^ CNS infiltrating leukocytes (F) in brain and spinal cord tissues. **Fig S2.** Acute DTx treatment in CX3CR1^Cre−ER^:R26^iDTR^ mice does not induce neurotoxic effects in the non-diabetic CNS. (A) Experimental design to validate the feasibility of depleting CNS-resident microglia without affecting peripheral *CX3CR1*-expressing immune cells. Four weeks following *Cre* recombinase induction with TAM (after the 5^th^ TAM injection), CX3CR1^Cre−ER^:R26^iDTR^ mice were administered 25 ng/g diphtheria toxin (DTx) once daily for 3 days. Tissue collection occurred 24 h after the last DTx injection. Control mice received PBS instead of DTx. (B) Gating strategies to identify CD11b^+^CD45^Hi^ blood leukocytes. (D) Confocal images of the primary visual cortex for Iba1^+^ (green), NeuN^+^ (red) and DAPI^+^ nuclei (blue). E–F, Quantification of Iba1^+^ cells/mm^3^ (E) and NeuN^+^ cells/mm^3^ (F) in the primary visual cortex of PBS and DTx treated CX3CR1^CreER^:R26^iDTR^ mice. (G) Confocal images of the retina for GFAP^+^ (magenta) and DAPI^+^ nuclei (blue). (H) Quantification of GFAP^+^ percent immunoreactive area. Data show mean ± SD, *n* = 6 to 9 mice per group where each dot represents an individual mouse. **P < 0.01, using Student’s *t*-test, with Welch’s correction. **Fig S3. **DTx treatment in diabetic CX3CR1^Cre−ER^:R26^iDTR^ mice targets CX3CR1^+^ cells in the CNS and does effect CX3CR1^+^ cells in the periphery. (A) Gating strategy to identify CD45^Hi^CD11b^+^SSC^Hi^ neutrophils, CD45^Hi^CD11b^+^SSC^Lo^ macrophages, CD45^Hi^CD11b^+^Ly6C^Lo^ tissue resident macrophages, CD45^Hi^CD11b^+^Ly6C^Hi^ inflammatory macrophages, CD45^Hi^CD11b^–^CD11c^+^ conventional dendritic cells and CD45^Hi^CD11b^+^CD11c^+^ myeloid-derived dendritic cells in blood leukocytes. B-D, Quantification of Bio-Plex Cytokine 23-plex analysis for cytokine and chemokine in serum isolates. (E) Gating strategy to identify CNS resident microglia CD11b^+^CD45^Lo^P2RY12^+^Ly6C^–^, monocyte-derived microglia CD11b^+^CD45^Lo^P2RY12^+^Ly6C^+^ and CD11b^+^CD45^Hi^ CNS infiltrating leukocytes. F–H, Flow cytometric quantification of CD11b^+^CD45^Lo^P2RY12^+^Ly6C^–^ CNS resident microglia (F), CD11b^+^CD45^Lo^P2RY12^+^Ly6C^+^ monocyte-derived microglia (G), and CD11b^+^CD45^Hi^ CNS infiltrating leukocytes (H). Data show mean ± SD, *n* = 4 to 6 mice per group where each dot represents an individual mouse. *P < 0.05, **P < 0.01, ***P < 0.001 ****P < 0.0001 using Student’s *t*-test, with Welch’s correction. **Fig S4.** Prolonged DTx exposure and recovery does not alter retinal pathology in the non-diabetic CX3CR1^Cre−ER^:R26^iDTR^ retina. (A) Experimental design for two-weeks DTx treatment in non-diabetic CX3CR1^Cre−ER^:R26^iDTR^ mice. Mice received citrate buffer, 2-weeks after TAM administration. At 6–8 weeks post citrate buffer treatment, mice received 3 daily doses of 25 ng/g DTx, followed by 1 dose of 25 ng/g DTx every 48 h for a total of 2-weeks. Tissues were collected after a 2-weeks recovery period. Control non-diabetic mice were administered PBS instead of DTx. (B) Confocal images of Iba1 (green) and NeuN-RBPMS (red). C-D, Quantification of Iba1^+^ cells/mm^3^ (C) and NeuN^+^RBPMS^+^ cells/mm^3^ (D). (E) Representation of cellular tracings for transformation index quantification. (F) Transformation index in non-diabetic, PBS and DTx treated mice, *n* = 98 to 150 microglia per group where each dot represents an individual microglia cell and bars show mean ± SD. (G) Confocal images of TUJ1 (turquoise) and GFAP (magenta). H-I, Quantification of percent immunoreactive area of TUJ1^+^ (H) and GFAP^+^ (I). (J) Confocal images of CD31 (red) and fibrinogen (white). K-L, Quantification of percent immunoreactive area of CD31^+^ (K) and fibrinogen^+^ (L). Data show mean ± SD, *n* = 6 to 9 mice per group where each dot represents an individual mouse (C, D, H, I, K, L). Data are mean ± SD, *n* = 120 to 180 microglia per group where each dot represents an individual microglia cell from *n* = 4–10 mice (E). *P < 0.05, **P < 0.01, ***P < 0.001 ****P < 0.0001 using Student’s *t*-test, with Welch’s correction. **Fig S5.** The effects of two-weeks DTx recovery on the CNS in CX3CR1^Cre−ER^:R26^iDTR^ mice. (A) Experimental design for two-weeks DTx treatment in non-diabetic and diabetic CX3CR1^Cre−ER^:R26^iDTR^ mice. Diabetes was induced via streptozotocin (STZ) in CX3CR1^CreER^:R26^iDTR^ mice 2-weeks after the last TAM injection. Control mice received citrate buffer as a vehicle control. 6–8 weeks after the last STZ (or citrate buffer control) injection, mice received 3 daily doses of 25 ng/g DTx, followed by 1 dose of 25 ng/g DTx every 48 h for a total of 2-weeks. Tissues were collected after a 2-weeks recovery period following the last DTx injection. Control non-diabetic and diabetic mice were administered PBS instead of DTx. (B) Gating strategy to identify CNS resident microglia CD11b^+^CD45^Lo^P2RY12^+^Ly6C^–^, monocyte-derived microglia CD11b^+^CD45^Lo^P2RY12^+^Ly6C^+^ and CD11b^+^CD45^Hi^ CNS infiltrating leukocytes. C-E, Flow cytometric quantification of CD11b^+^CD45^Lo^P2RY12^+^Ly6C^–^ CNS resident microglia (C), CD11b^+^CD45^Lo^P2RY12^+^Ly6C^+^ monocyte-derived microglia (D), and CD11b^+^CD45^Hi^ CNS infiltrating leukocytes (E). Data show mean ± SD, *n* = 4 to 6 mice per group where each dot represents an individual mouse. *P < 0.05, **P < 0.01, ***P < 0.001 ****P < 0.0001 using Student’s *t*-test, with Welch’s correction. **Fig S6.** Differences in microglia depletion efficiencies in the CX3CR1^CreER^:R26^iDTR^ model compared to PLX-5622 model. (A) Experimental design to compare microglia depletion efficiency in CX3CR1^CreER^:R26^iDTR^ model and PLX-5622 model of microglia depletion. Diabetic CX3CR1^CreER^:R26^iDTR^ mice were DTx treated for 2-weeks and in diabetic CX3CR1-WT mice were PLX-5622 treated for 2-weeks. Control diabetic CX3CR1^CreER^:R26^iDTR^ mice were given PBS and control diabetic CX3CR1-WT mice remained on normal chow. Tissues were collected immediately after the 2-weeks treatment regimen. (B) Flow cytometric quantification of CD11b^+^CD45^Lo^Zombie^–^ microglia. C-D, Quantification of the number of Iba1^+^ cells/mm^3^ (C) and transformation index, where *n* = 98 to 150 microglia per group where each dot represents an individual microglia cell and bars show mean ± SD (D). Data show mean ± SD, *n* = 4 to 10 mice per group where each dot represents an individual mouse (B, C). Data are mean ± SD, *n* = 52 to 150 microglia per group where each dot represents an individual microglia cell from *n* = 4–10 mice (D). *P < 0.05, **P < 0.01, ***P < 0.001 ****P < 0.0001 using Student’s *t*-test, with Welch’s correction. **Fig S7. **Transcriptional profile changes associated with the glial limitans. (A) Experimental design to pharmacologically deplete and repopulate microglia using PLX-5622 for retinal mRNAseq analysis. Six weeks following STZ-induced diabetes, CX3CR1-WT mice were fed PLX-5622 for two weeks, followed by a 2-weeks recovery period. Diabetic control mice were fed normal chow. B-G, Graphical analysis of the fold change in gene expression for *Gfap* (B), *Claudin-1* (C), *Claudin-4* (D), *F11r* (E), *Adamts13* (F) and *Csf3* (G). **Fig S8.** Principal component analysis plots of diabetic samples and non-diabetic samples. Principal component analysis plots to show biological replicate clustering by sample type for non-diabetic *n* = 6 (green) versus 6-weeks diabetic *n* = 5 (blue) (A), non-diabetic *n* = 6 (green) versus 8-weeks diabetic normal chow *n* = 5 (blue) (B), non-diabetic *n* = 6 (green) versus 8-weeks diabetic PLX-5622 chow treated *n* = 4 (blue) (C), non-diabetic *n* = 6 (green) versus 10-weeks diabetic normal chow recovery *n* = 5 (blue) (D) and non-diabetic *n* = 6 (green) versus 10-weeks diabetic PLX-5622 chow recovery *n* = 5 (blue) (E). **Fig S9**. Principal component analysis plots of diabetic normal chow versus diabetic PLX-5622 chow samples. Principal component analysis plots to show biological replicate clustering by sample type for 8-weeks diabetic PLX-5622 chow treated *n* = 4 (green) versus 8-weeks diabetic normal chow *n* = 5 (blue) (A) and 10-weeks diabetic PLX-5622 chow recovery *n* = 5 (green) versus 10-weeks diabetic *n* = 5 (blue) (B).

## Data Availability

The datasets used and/or analyzed during the current study are available from the corresponding author on reasonable request.

## Abbreviations

AMD: Age-related macular degeneration
TUJ1: β Tubulin III
BBB: Blood brain barrier
BRB: Blood retinal barrier
CNS: Central nervous system
DAMPs: Danger associated molecular patterns
DCs: Dendritic cells
DR: Diabetic retinopathy
DEGs: Differently expressed genes
DTx: Diphtheria toxin
DTR: Diphtheria toxin receptor
GFAP: Glial fibrillary acidic protein
HBSS: Hank’s balanced salt solutions
IF: Immunofluorescent
i.p: Intra-peritoneal
Iba1: Ionized calcium binding adaptor molecule-1
JAMs: Junctional adhesion molecules
LPS: Lipopolysaccharide
NeuN: Neuronal nuclei
NVU: Neurovascular unit
ND: Non-diabetic
NC: Normal chow
CD31: Pecam-1
PFA: Paraformaldehyde
PVC: Primary visual cortex
PCA: Principal component analysis
PIC: Proteinase inhibitor cocktail
RNFL: Retinal nerve fiber layer
RBPMS: RNA-binding protein with multiple splicing
siRNA: Small interfering RNA
STZ: Streptozotocin
TAM: Tamoxifen
TdT: TdTomato
TJ: Tight junction
TI: Transformation index
wk(s): Week
QC: Quality control
