# Supplementary material for: Models of microglia depletion and replenishment elicit protective effects to alleviate vascular and neuronal damage in the diabetic murine retina
Source: J Neuroinflammation. 2022 Dec 14;19:300. doi: 10.1186/s12974-022-02659-9 (PMC9753268; doi:10.1186/s12974-022-02659-9)

Supplementary Figure 1

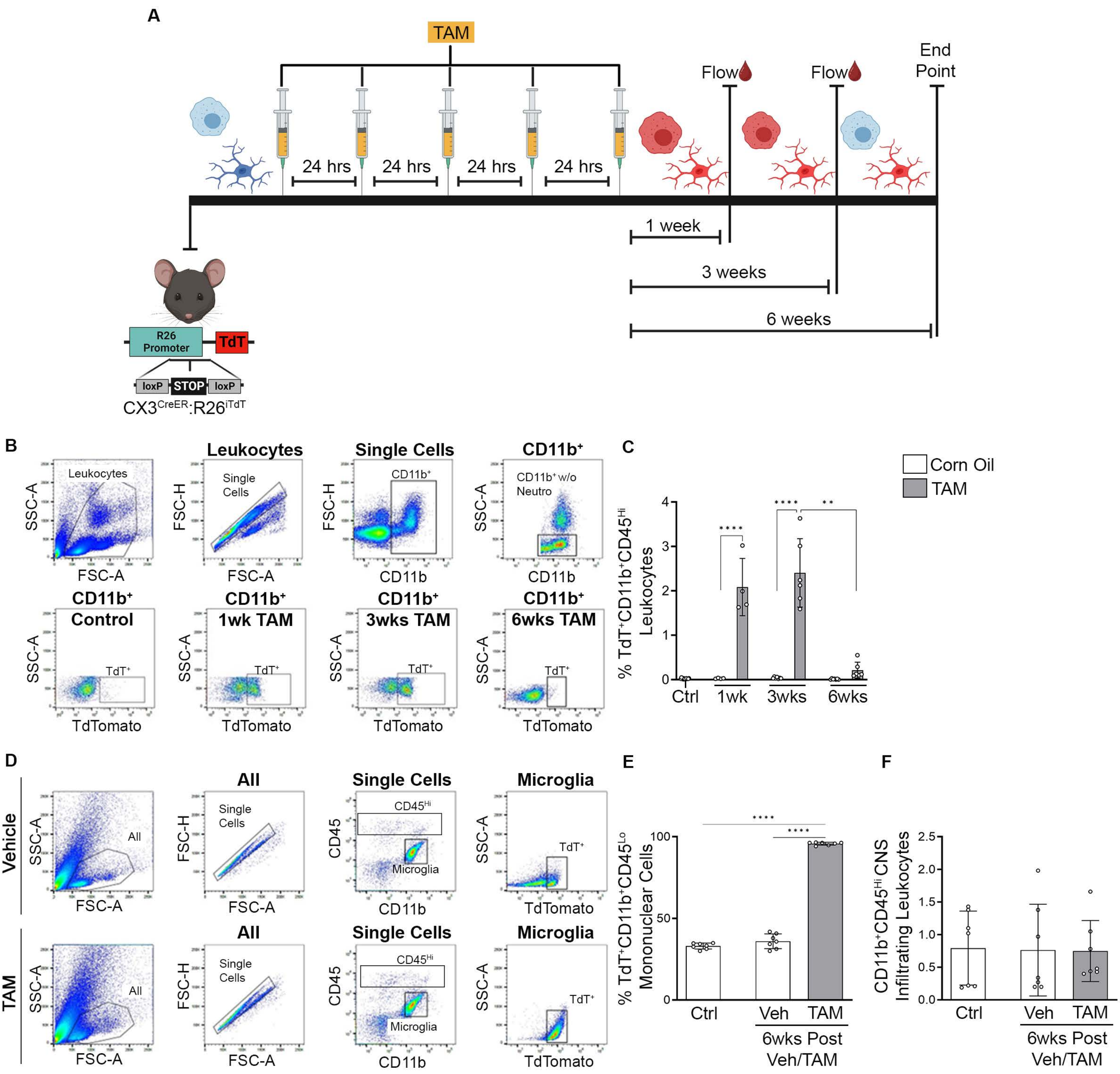

Supplementary Figure 2

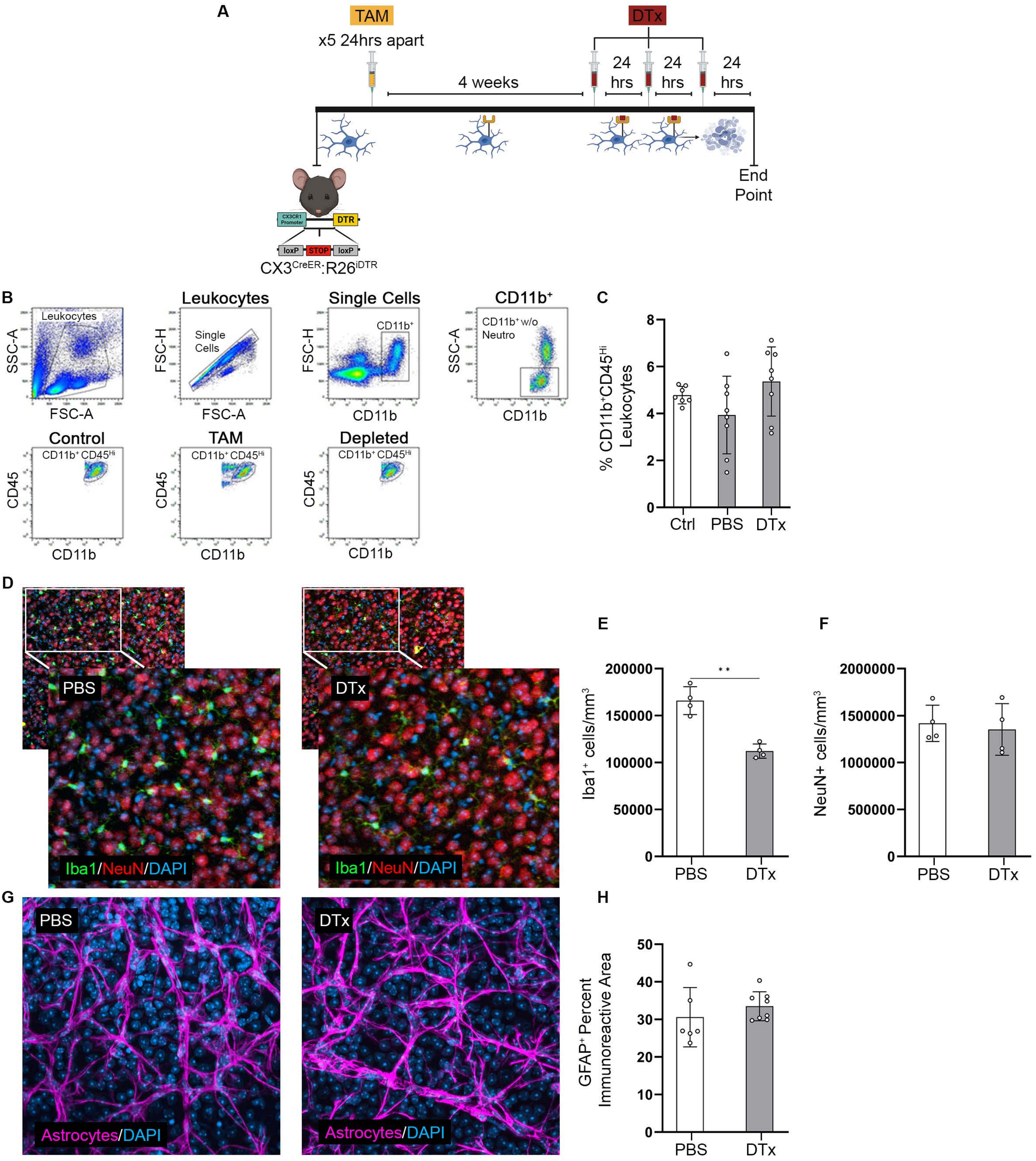

Supplementary Figure 3

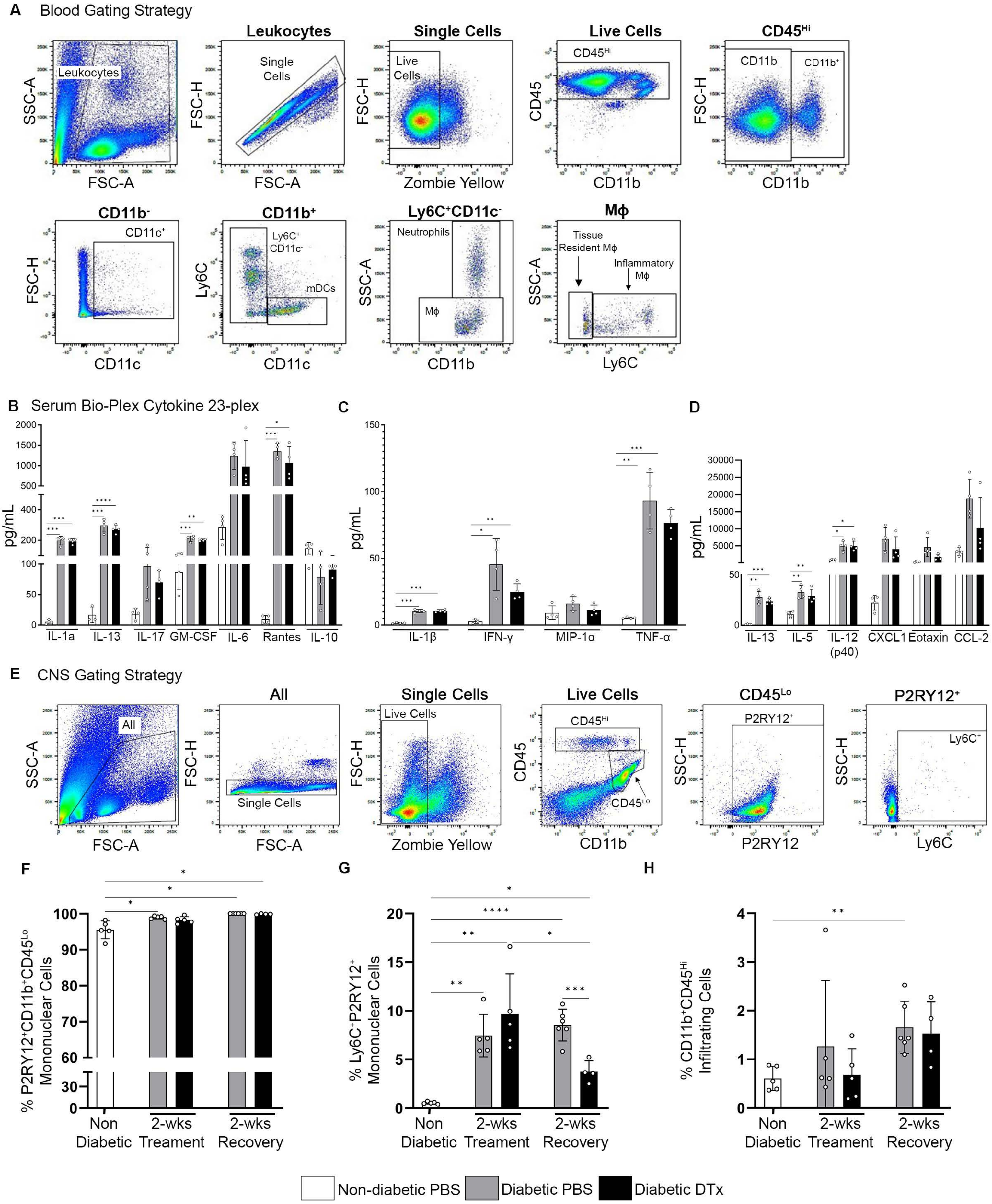

Supplementary Figure 4

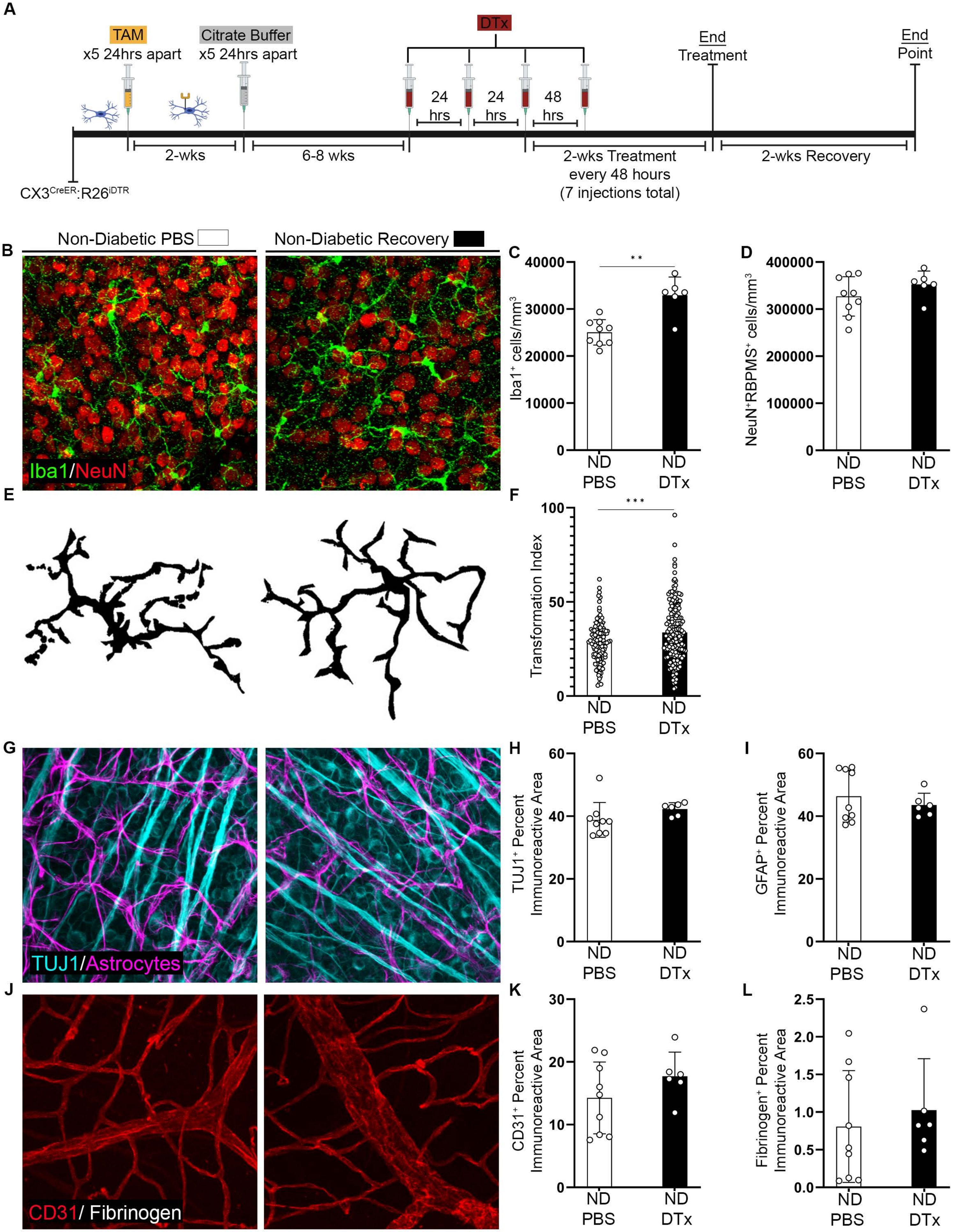

Supplementary Figure 5

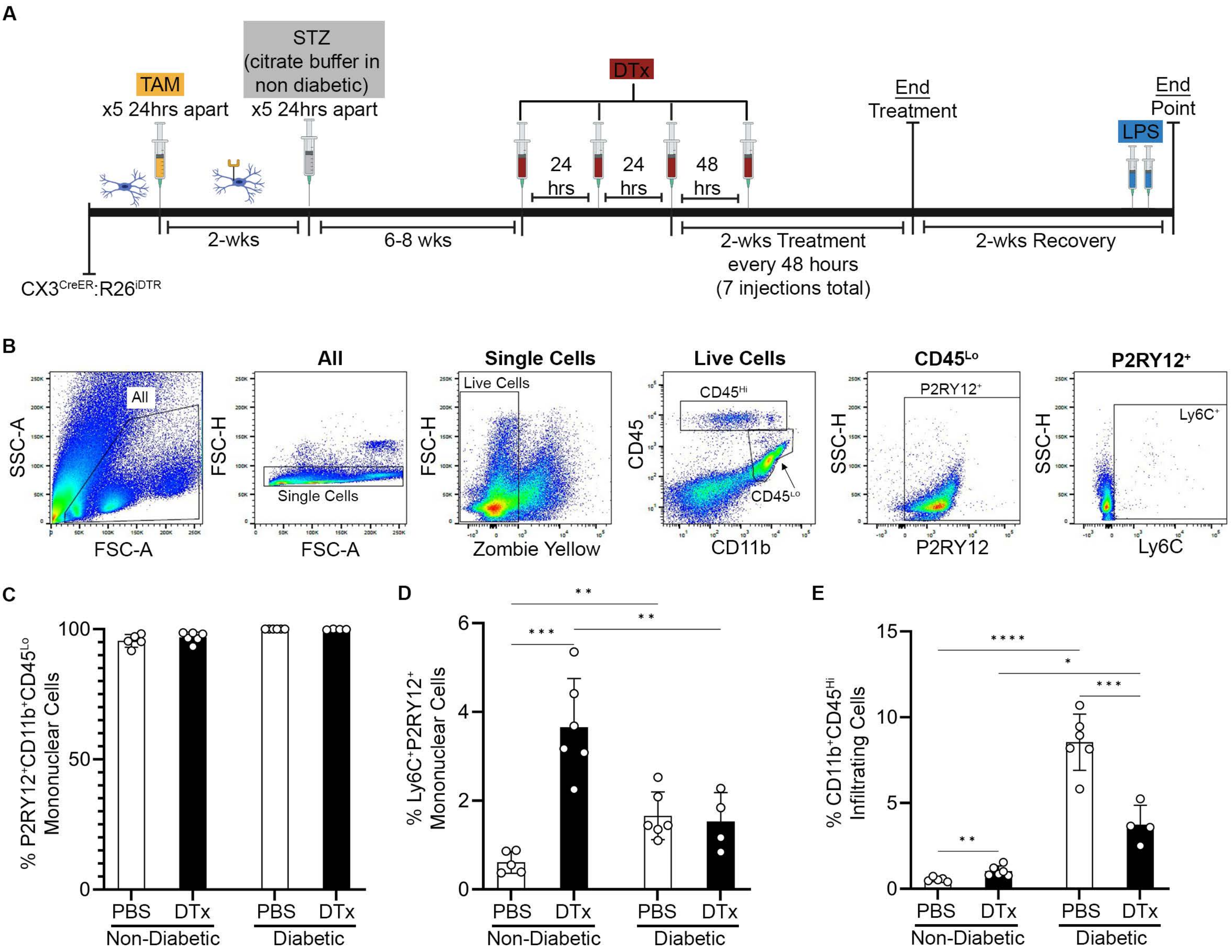

Supplementary Figure 6

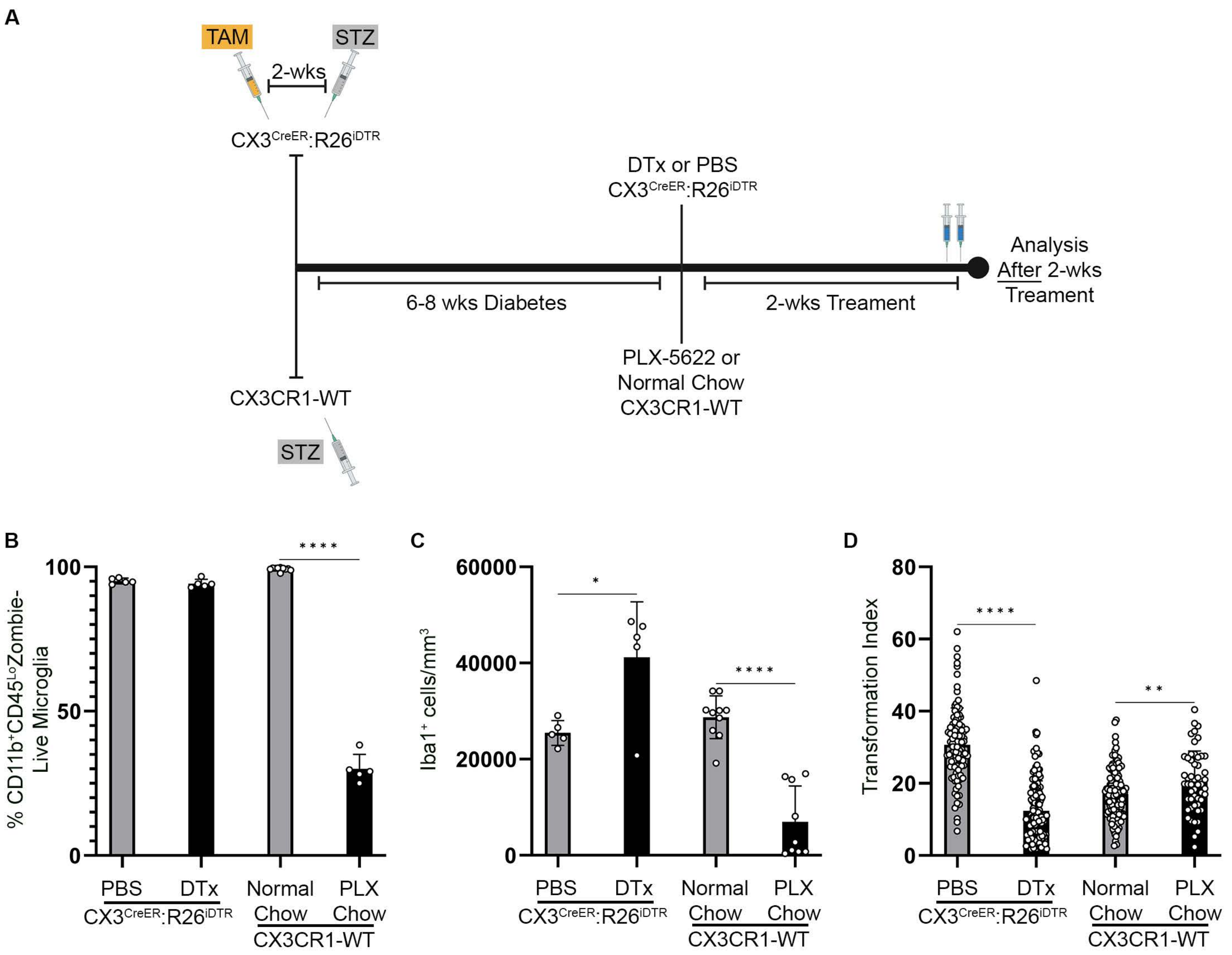

Supplementary Figure 7

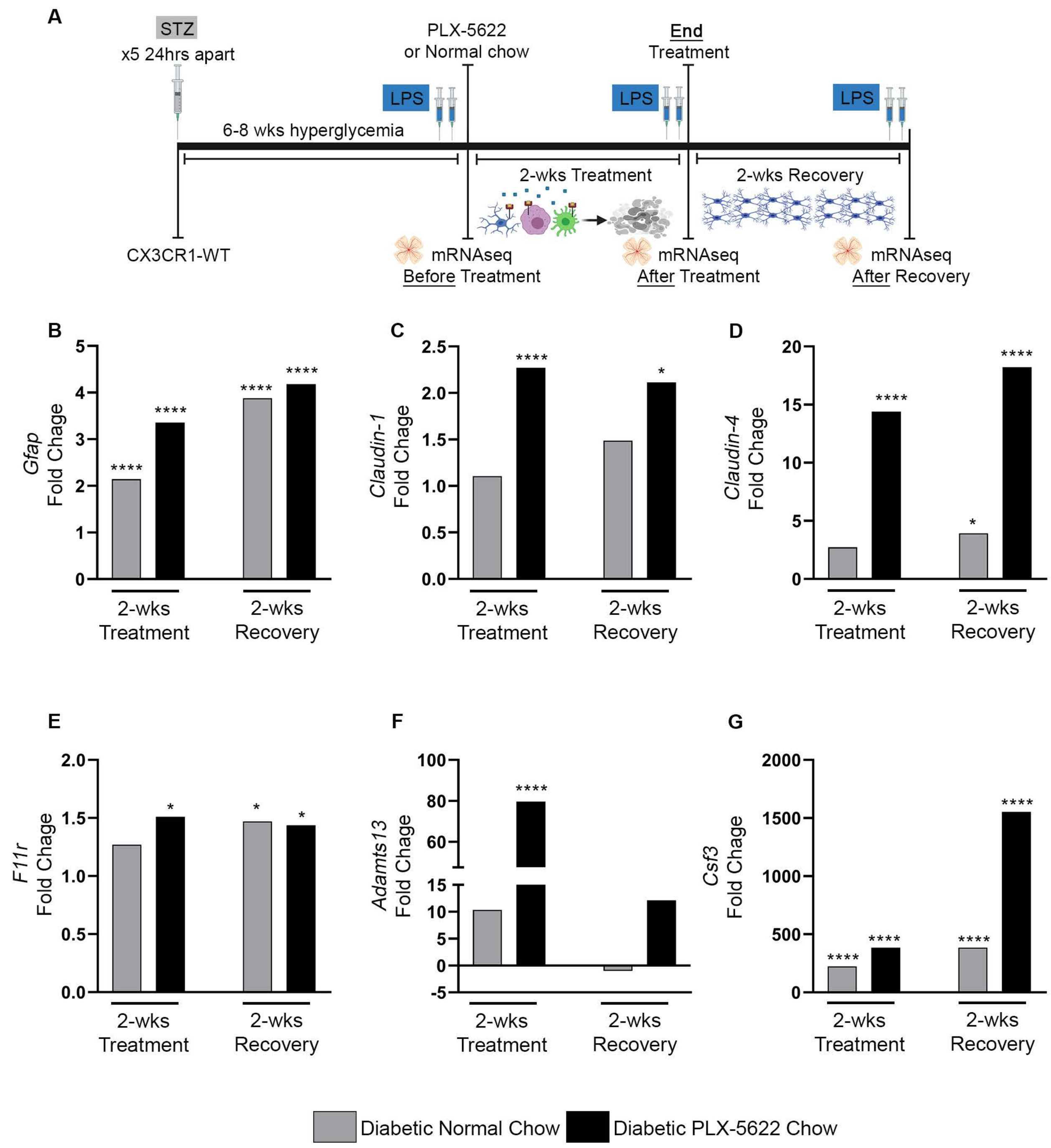

Supplementary Figure 8

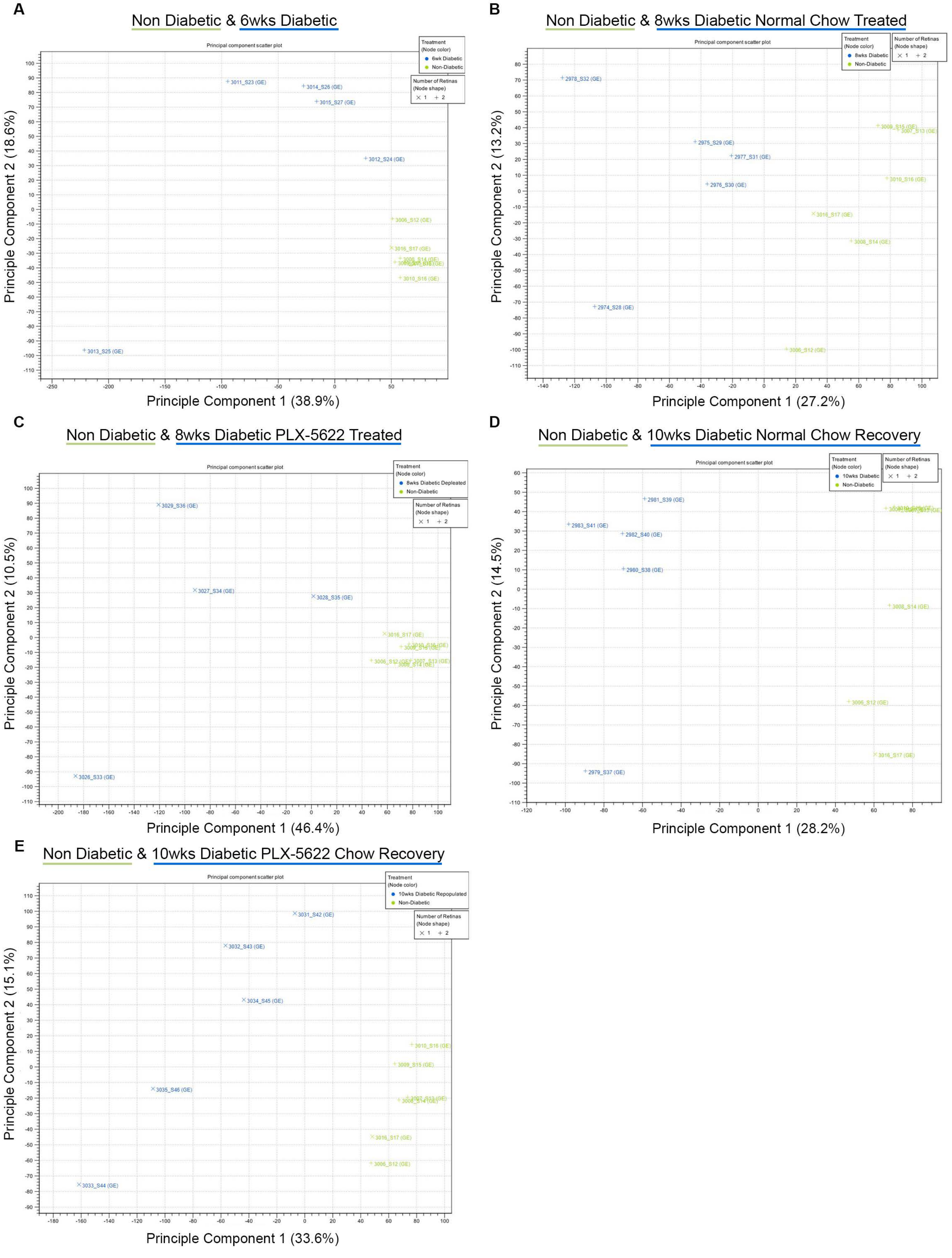

Supplementary Figure 9

A

8wks Diabetic PLX-5622 Treated &
8wks Diabetic Normal Chow Treated

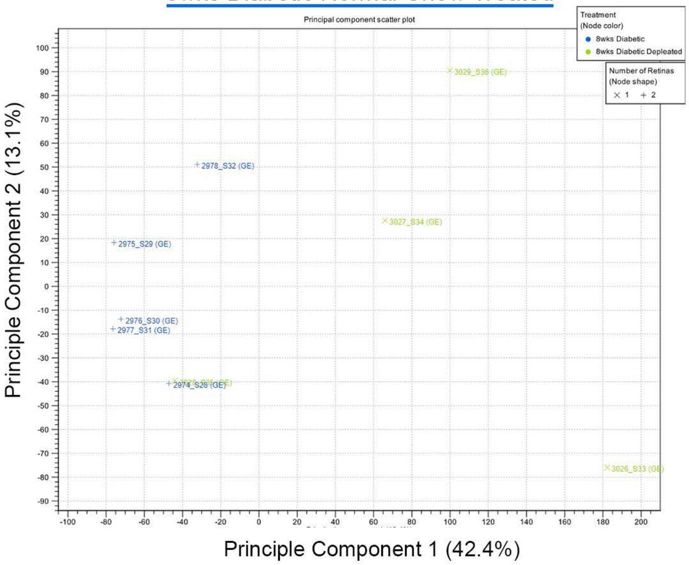

B

10wks Diabetic PLX-5622 Recovery &
10wks Diabetic Normal Chow Recovery

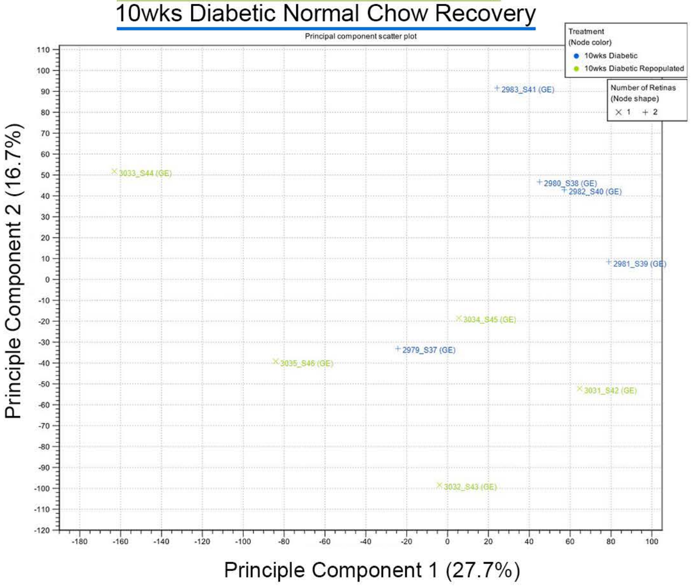

Supplement: Supplementary file 1 — Additional file 1: Fig S1. Characterizing CX3CR1CRE−ER expression modulated by TAM in CX3CreER:R26iTdT mice. (A) Experimental design to confirm that Cre penetrance targets CX3CR1-expressing cells in the retina and brain without affecting peripheral CX3CR1-expressing immune cells. CX3CreER:R26iTdT mice were injected once daily for 5 days with tamoxifen (TAM). One week and 3-weeks after the last TAM injection, flow cytometric analysis was performed on blood leukocytes to track the percentage of TdT+CD11b+CD45Hi leukocytes. At six-weeks post TAM administration, tissues were collected for flow cytometric and immunohistochemical analysis. (B) Gating strategy to identify TdT+CD11b+CD45Hi blood leukocytes. (C) Graphical representation of flow cytometric quantification of TdT+CD11b+CD45Hi blood leukocytes. (D) Gating strategy to identify TdT+CD11b+CD45Lo microglia in brain and spinal cord tissues. E–F, Graphical representation of flow cytometric quantification of TdT+CD11b+CD45Lo microglia (E) and TdT+CD11b+CD45Hi CNS infiltrating leukocytes (F) in brain and spinal cord tissues. Fig S2. Acute DTx treatment in CX3CR1Cre−ER:R26iDTR mice does not induce neurotoxic effects in the non-diabetic CNS. (A) Experimental design to validate the feasibility of depleting CNS-resident microglia without affecting peripheral CX3CR1-expressing immune cells. Four weeks following Cre recombinase induction with TAM (after the 5th TAM injection), CX3CR1Cre−ER:R26iDTR mice were administered 25 ng/g diphtheria toxin (DTx) once daily for 3 days. Tissue collection occurred 24 h after the last DTx injection. Control mice received PBS instead of DTx. (B) Gating strategies to identify CD11b+CD45Hi blood leukocytes. (D) Confocal images of the primary visual cortex for Iba1+ (green), NeuN+ (red) and DAPI+ nuclei (blue). E–F, Quantification of Iba1+ cells/mm3 (E) and NeuN+ cells/mm3 (F) in the primary visual cortex of PBS and DTx treated CX3CR1CreER:R26iDTR mice. (G) Confocal images of the retina for GFAP [file 12974_2022_2659_MOESM1_ESM.pdf]
